# Smooth muscle cell-specific *Notch1* haploinsufficiency restricts the progression of abdominal aortic aneurysm by modulating CTGF expression

**DOI:** 10.1371/journal.pone.0178538

**Published:** 2017-05-31

**Authors:** Jaspreet Sachdeva, Advitiya Mahajan, Jeeyun Cheng, Jeremy T. Baeten, Brenda Lilly, Helena Kuivaniemi, Chetan P. Hans

**Affiliations:** 1 Ohio State University, Columbus, Ohio, United States of America; 2 Cardiology, Medical Pharmacology & Physiology and Dalton Cardiovascular Research Center, University of Missouri, Columbia, Missouri, United States of America; 3 Center for Cardiovascular Research and The Heart Center, Nationwide Children's Hospital, Columbus, Ohio, United States of America; 4 Division of Molecular Biology and Human Genetics, Department of Biomedical Sciences, Faculty of Medicine and Health Sciences, Stellenbosch University, Tygerberg, South Africa; Max Delbruck Centrum fur Molekulare Medizin Berlin Buch, GERMANY

## Abstract

**Aims:**

Infiltration of macrophages and apoptosis of vascular smooth muscle cells (VSMCs) promote the development of abdominal aortic aneurysm (AAA). Previously, we demonstrated that global Notch1 deficiency prevents the formation of AAA in a mouse model. Herein, we sought to explore the cell-specific roles of Notch1 in AAA development.

**Methods and results:**

Cell-specific *Notch1* haploinsufficient mice, generated on *Apoe*^*-/-*^ background using Cre-lox technology, were infused with angiotensin II (1000 ng/min/kg) for 28 days. *Notch1* haploinsufficiency in myeloid cells (n = 9) prevented the formation of AAA attributed to decreased inflammation. Haploinsufficiency of *Notch1* in SMCs (n = 14) per se did not prevent AAA formation, but histoarchitectural traits of AAA including elastin degradation and aortic remodeling, were minimal in *SMC-Notch1*^*+/-*^*;Apoe*^*-/-*^ mice compared to *Apoe*^*-/-*^ mice (n = 33). Increased immunostaining of the contractile SMC-phenotype markers and concomitant decreased expression of synthetic SMC-phenotype markers were observed in the aortae of *SMC-Notch1*^*+/-*^*;Apoe*^*-/-*^ mice. Expression of connective tissue growth factor (CTGF), a matrix-associated protein that modulates the synthetic VSMC phenotype, increased in the abdominal aorta of *Apoe*^*-/-*^ mice and in the adventitial region of the abdominal aorta in human AAA. *Notch1* haploinsufficiency decreased the expression of Ctgf in the aorta and *in vitro* cell culture system. *In vitro* studies on SMCs using the Notch1 intracellular domain (NICD) plasmid, dominant negative mastermind-like (dnMAML), or specific siRNA suggest that Notch1, not Notch3, directly modulates the expression of CTGF.

**Conclusions:**

Our data suggest that lack of Notch1 in SMCs limits dilation of the abdominal aorta by maintaining contractile SMC-phenotype and preventing matrix-remodeling.

## Introduction

Abdominal aortic aneurysm (AAA) is a localized dilation of the abdominal aorta exceeding the normal diameter by more than 50% of its original size[1, 2]. The prevalence of AAA ranges from 1.3% in men 45 to 54 years of age to ~9% in the population over 65 years of age[2, 3]. In the United States, aortic aneurysms account for more than 10,000 deaths per year[2]. Understanding the underlying mechanisms of AAA expansion will lead to novel pharmacologic therapies to reduce AAA development and rupture[4–6].

The development of AAA is characterized by transmural infiltration of macrophages and depletion of vascular smooth muscle cells (VSMCs) at the vascular injury site, which results in the focal enlargement of the abdominal aorta[7–10]. Excessive infiltration of macrophages and apoptosis of VSMCs stimulates activation of matrix metalloproteinases (MMPs) resulting in the fragmentation of the elastin and interstitial collagenous matrix of the medial layer of the aorta[11]. VSMCs are capable of phenotypic modulation in response to different environmental cues. Contractile and synthetic VSMCs, which represent the two ends of a spectrum, have different morphological components and functional roles[12]. Contractile VSMCs are quiescent cells, display an elongated spindle-shaped morphology and express a unique repertoire of contractile proteins that serve as VSMC markers including SMA- α, SM22α, smMHC and smoothelin[13, 14]. Vascular injury, results in abundant proliferative and migratory phenotypic modulation of VSMCs, which produce extracellular matrix (ECM) proteins such as elastin and collagen to provide tensile strength to the injured vessel wall. However, enhanced degradation of structural proteins, together with a reduced capacity to synthesize new matrix proteins, progressively weaken the aortic wall, resulting in rupture.

Notch1 signaling has gained substantial interest in the past few years because of its involvement in multiple aspects of vascular development and remodeling including myeloid and VSMC differentiation[15–23]. Notch1 signaling also regulates vascular inflammation[24], VSMC apoptosis[25, 26], and MMP-dependent proteolysis[27, 28]. Many of these pathways are implicated in the pathogenesis of aneurysms[29]. There are four types of Notch receptors (Notch1-4) expressed in all vascular cells including endothelial cells, VSMCs and macrophages[30]. Upon ligand receptor binding (Jagged or Delta-like), the Notch intracellular domain (NICD; active form of Notch1) is released from the membrane and is translocated into the nucleus where it binds to the transcriptional regulator RBPJ (also known as CBF-1). NICD activates the transcription of target genes by displacing the repressor complex from CBF-1 genes and recruiting activators including mastermind-like protein (MAML)[31]. Our laboratory identified Notch1 signaling as an important factor that mediates inflammatory response and disease initiation in angiotensin II (AngII) induced mouse model of AAA[32, 33]. We also reported that reconstitution of bone marrow-derived cells from *Notch1*^*+/-*^*;Apoe*^*-/-*^ mice (donor) in irradiated *Apoe*^*-/-*^ mice (recipient) decreased the occurrence of aneurysm[33]. The diversified roles of Notch1 in various vascular cells demonstrate the complexity of Notch signaling and warrant studying the individual contributions of the Notch1 receptor in each vascular cell type on aneurysms.

The goal of this study was to define the unique contributions of SMC-specific and myeloid-specific Notch1 signaling in the development of AAA. We present evidence that myeloid-specific *Notch1* haploinsufficiency protects against the formation of AAA, whereas SMC-specific *Notch1* haploinsufficiency interferes with the progression of aortic aneurysms. Collectively, these findings imply that Notch1 signaling in different cell types affects the development of aortic aneurysm by multifarious mechanisms and represents a potential target to mitigate the deleterious effects of AAA at different stages of the disease progression.

## Materials and methods

To determine the contributions of each vascular cell type in AAA development, we used a Cre-Lox recombination approach. The following cell-specific *Notch1* haploinsufficient mice were generated:

1. Myeloid specific *Notch1* haploinsufficient mice *(LysM-Cre)*.
2. Smooth muscle cell (SMC) specific *Notch1* haploinsufficient mice *(smMHC-Cre)*.
3. Endothelial cell (EC) specific *Notch1* haploinsufficient mice *(Tie2-Cre)*.

The *LysM-Cre*, *smMHC-Cre*, and *Tie2-Cre* mice were purchased from Jackson lab (Ann Arbor). Cell specific *Notch1* haploinsufficient mice were generated by crossing the offspring of *Notch1*^*flox/WT*^*;Apoe*^*-/-*^ female mice with their respective *Cre* male mice against *Apoe*^*-/-*^ background as explained in S1 Table. All animals were genotyped by polymerase chain reaction (PCR) as shown in S2 and S3 Tables). Aneurysmal studies were performed on these mice by infusing AngII or saline for 28 days using published protocols[32, 33]. Briefly, mice were anesthetized in a closed chamber with 3% isoflurane in oxygen for 2 to 5 minutes until immobile. Each mouse was then removed, and taped on a heated (35–37°C) procedure board with 1.0–1.5% isoflurane administered via nosecone during minor surgery. After 28 days, mice were deep anesthetized with ketamine/xylazine (100 and 20 mg/kg, respectively) the aortae were dissected, fixed in 10% formalin, and maximal aortic diameters measured. The animal experiments were approved by Institutional Animal Care and Use Committee at the Research Institute at Nationwide Children’s Hospital. All the animal experiments conform the NIH guidelines (Guide for the care and use of laboratory animals).

### Isolation, culture, flow cytometry and co-culture of primary abdominal aorta-derived vascular smooth muscle cells (VSMCs) and bone marrow derived macrophages

Primary abdominal aorta-derived VSMCs were isolated from *WT*, *Notch1*^*+/-*^, *Apoe*^*-/-*^ or *Apoe*^*-/-*^*;Notch1*^*+/-*^ mice (6–8 weeks of age). Briefly, mice were sacrificed with CO_2_ asphyxiation and perfused with ice cold PBS through the left ventricle. Abdominal aorta was isolated and cleared from exogenous fat and adventitial layer followed by enzymatic digestion using collagenase/elastase (1 mg/mL collagenase, cat# LS004174, 0.744 units/mL elastase, cat# LS002279; Worthington) as described[34, 35]. VSMCs were grown in DMEM/F12 media without phenol red with 2% penicillin/streptomycin, 1% fungizone (Invitrogen), and 20% fetal bovine serum (Tissue Biologics) until cells were confluent, and then trypsinized (0.25%). Purity of SMC was confirmed by performing immunofluorescence on the isolated cells with antibodies to smooth muscle actin (SMA-α) as shown in our previous studies [35, 36]. All studies with VSMCs were performed on cells from passage 3–8. For apoptosis assay, VSMC were grown on 6 well plates. At ~70% confluence, the cells were treated with 1% FCS in DMEM containing lipopolysaccharide (LPS;100 ng/ml) for 24 hours. Afterwards, cells were stained with Annexin V Apoptosis Detection Kit (eBiosciences) using standard protocols. Flow cytometry was performed on a Becton Dickinson LSRII flow cytometer with DiVa software and data were analyzed with Flow Jo software (Ashland, OR) to quantify the percentage of differential apoptotic population. For co-culture assay, the bone marrow–derived macrophage (BMDMs) were isolated from *Notch1*^*+/-*^ and wild-type (*WT)* mice and differentiated to naïve macrophages onto transwell chambers using the standard protocol[32, 33]. The cells were then pretreated with vehicle or LPS (100 ng/ml) and interferon‐γ (IFN-γ; 20 ng/ml) for 3h, as described [37]. After pretreatment, the transwell chambers containing BMDMs were washed with media to remove excess of LPS/IFN-γ, and were transferred to another plate in which WT SMCs were previously grown at the ratio of 1:1. After 24 hours, the SMCs were washed and RNA was isolated for further experiments.

### Transfection of smooth muscle cells

Plasmids and siRNA sequences were used for transfecting the cells. NICD and dnMAML plasmids were obtained from Dr. Lilly’s laboratory. Predesigned Notch1 (s70698, Invitrogen) and Notch3 (s70707, Invitrogen) siRNA were used with Lipofectamine 3000 (L3000, Invitrogen) and Lipofectamine RNA iMAX (13778, Invitrogen). The cells were plated into 6-, 12-, or 24-well plates with their normal growth serum. Two hours prior to the transfection, DMEM media without serum and antibiotics were added to the cells. The reagents for transfection were prepared using OptiMEM media (31985–070, Invitrogen). Two different tubes were prepared for each well; one containing the OptiMEM and Lipofectamine P3000 and the other containing OptiMEM, Lipofectamine P3000/RNA iMAX reagent, and either the plasmid (2μl/μg DNA) or the siRNA. These two tubes were then combined and the mixture was incubated for 5 minutes at room temperature. This mixture was then added directly onto the cells containing the DMEM serum-free and antibiotic-free media. The media was changed four to six hours after transfection with normal growth medium.

### Human aortic tissue samples

After written informed consent, full thickness infrarenal abdominal aortic wall tissue samples were collected from patients who underwent repair operations for AAA (n = 3, white men aged 67, 70, and 72 years) at the Harper University Hospital, Detroit, Michigan. Non-aneurysmal control samples were collected from the infrarenal segment of aorta (n = 3; white men aged 53, 53, and 78 years) at autopsies within 24 hours of death at the Detroit Coroner’s Office. The samples were considered discarded tissue samples and no consent was required for the autopsy samples. At the time of harvesting, all samples were coded and no link to personal identifying information was kept. Tissue samples were incubated in phosphate-buffered formalin and embedded in paraffin for histological and immunohistochemical analyses. The collection of the tissue samples and their use for research projects was approved by the Institutional Review Board of Wayne State University, Detroit, Michigan, and the research carried out was in compliance with the Helsinki Declaration[32].

### Histology, immunostaining, and quantitation

The mice from the experiments were euthanized and aortae were dissected. The thoracic and abdominal aortae were fixed in 10% formalin and serial sections were obtained. The abdominal aorta was cut into two equal halves and serial sections of the abdominal aorta (5μM) were prepared throughout the aneurysmal area of the abdominal aorta. Several sections of the abdominal aorta at regular intervals (200μM) were subjected to hematoxylin and eosin (H&E), elastin, and Masson’s trichrome stain for histoarchitectural evaluation of aneurysm. The serial tissue sections obtained from these mice were further subjected to immunohistochemistry (IHC) or immunofluorescence (IF). For IHC, the abdominal aortae were stained with antibodies for active caspase-3, matrix metallopeptidase-2 (Mmp2), Mmp9, Mmp12, smooth muscle myosin heavy chain (smMHC), alpha-smooth muscle actin (SMA- α), osteopontin, vimentin, connective-tissue growth factor (Ctgf), NICD, interferon regulatory factor 8 (Irf8), Cd206, and monocyte chemotactic protein-1 (Mcp1). The intensity of the immunostaining was evaluated by obtaining 4–5 images from random areas of interest at 40X from each tissue (n = 4) and quantified using ‘Fiji’ version of Image J following the software directions[38]. For IF, CTGF and anti-fibroblast TE-7 were used to co-stain the sections of the human tissue. Antifade Mounting Medium containing DAPI (Vector Laboratories) was used to stain nuclei and mount the slides. The specificity of the antibody staining was determined using non-specific IgG against the source of host species. IHC and IF was performed using protocols described in a previous study[32, 33]. The list of antibodies can be found in S4 Table.

### Protein isolation and Western blot

Total proteins were extracted from the cells with RIPA buffer supplemented with protease and phosphatase inhibitor cocktails (Roche). After homogenization, protein concentrations were measured and 20 μg of protein were loaded onto 10% SDS-polyacrylamide gel, and transferred to PVDF membrane using standard protocols. The membranes were probed for CTGF and NICD using specific antibodies using standard protocol[35–37, 39]. GAPDH antibody was used for internal loading control.

### RNA isolation and qRT-PCR

Total RNA was isolated from the primary cell lines and the aortic tissue (suprarenal aorta approximately 5 mm thick) using TRIzol reagent (Ambion) according to manufacturer’s protocol. The aortic tissue samples were first homogenized using TissueLyser II (Qiagen). Isolated RNA underwent a DNAse treatment using DNAse Treatment and Removal reagent (Ambion) to remove contaminating genomic DNA. cDNA was synthesized using the SuperScript VILO kit (Invitrogen) and subjected to quantitative PCR (qPCR) by SYBR Green (Applied Biosystems) using an Applied Biosystems 7500 Fast Real-Time PCR System. The samples were run in triplicate and the fold-change was determined by normalizing CT values against *Rpl13a*. The primers are listed in S5 Table.

### Vascular smooth muscle cell contractility assay

The ability of cells to contract a collagen gel matrix was determined using a 96-well collagen gel contraction assay using standard protocol[40]. Aortic VSMCs isolated from *Apoe*^*-/-*^ or *Notch1*^*+/-*^*;Apoe*^*-/-*^ mice were first cultured in a 6-well plate and treated with or without 10 ng/ml CTGF for 72 hours. Cells were then trypsinized, counted, and suspended in a collagen solution at a concentration of 6x10^5^ cells/ml. This collagen solution contained the following: 43% (v/v) sterile PBS (Hyclone), 34% (v/v) 3 mg/ml rat tail collagen (BD Biosciences), 13.6% (v/v) serum-free DMEM (Hyclone), 7.6% (v/v) 10x Medium 199 (GIBCO), and 1% (v/v) FBS (Mediatech). The solution was brought to neutral pH (~7.0) by addition of NaOH using the phenol red indicator in the DMEM. Then, 70 μl of this collagen+cell solution was loaded into the wells of a 96-well plate and incubated at 37°C for 30 minutes to allow collagen to polymerize. A total of 140 μl of DMEM with 2% FBS was then added and the wells were incubated for 48 hours. The gels were then gently released from attachment to walls of the wells by Dumont #55 Forceps and 0 hour images were taken. The media was then replaced with 10% FBS DMEM to induce contraction and incubated for further 2 hours. After 2 hours, another image was taken of each well to determine the amount of gel contraction. Each treatment group was represented by 6 replicates in each experiment, and repeated in 3 independent experiments. The images were analyzed using ImageJ software[41] to measure the gel area at each time-point, from which the percentage decrease of gel area was determined[42].

### Aortic ring assay

Aortae from *Apoe*^*-/-*^ male mice were isolated. These aortae were dissected to obtain 4–5 sections (rings) from the abdominal region for transfection. The aortic rings were treated with the following in the absence or presence of CTGF (Invitrogen): NICD and dnMAML plasmid (along with the empty vector), non-specific siRNA (4390846, Invitrogen), siRNA Notch1 (s70700, Invitrogen), and siRNA Notch3 (s70708, Invitrogen) using standard protocol[43]. Plasmids and pre-designed siRNA were transfected with Lipofectamine 3000 and Lipofectamine RNAiMAX, respectively. The rings were plated onto chamber slides in DMEM High Glucose media (Hyclone) with 10% FBS (Fisher Scientific). Two hours prior to the transfection, DMEM media without serum and antibiotics were added to the chamber slides. The reagents for transfection were prepared using OptiMEM media (Invitrogen). Two different tubes were prepared for each well; one containing the OptiMEM and Lipofectamine 3000 or RNA iMAX and the other containing OptiMEM, P3000 reagent, and either the plasmid or the siRNA. These two tubes were then combined and the mixture was incubated for 5 minutes at room temperature. This mixture was the added directly onto the chamber slides with the aortic rings containing the DMEM serum-free and antibiotic-free media. The media was changed 24 hours after transfection to DMEM High Glucose media with 10% FBS. The aortic rings were then treated using recombinant human CTGF protein (PHG0286; Gibco) for additional 48 hours. The rings were then collected and processed for histology.

### Statistical analysis

Statistical comparisons were performed using either the Student t-test or 1-way ANOVA followed by the Bonferroni multiple comparison correction. GraphPad Prism version 5.0 (GraphPad Software, Inc., CA) was used for these comparisons and P<0.05 was considered significant. Assumptions of normality and equal variance were tested and satisfied using SAS software version 9.3 (SAS Institute Inc., NC). For multiple comparisons, a post-Bonferroni test with Holm correction (for 3 comparisons) was performed for maximal aortic width graph and survival graph. For the real-time RT-PCR quantification, we performed the Kruskal–Wallis test using a non-parametric method for the overall significance of the data and ordinary ANOVA followed by a Bonferroni-Holm multiple comparisons test with single-pooled variance for multiple comparisons using GraphPad Prism version 5.0. For the statistical analysis of actual incidence, Fisher’s exact test was used with the SAS software. The actual incidence of the disease is defined as 50% of greater increase in the maximal aortic width compared with the control group.

## Results

### Cell-specific *Notch1* haploinsufficiency differentially interferes with AAA development in AngII-induced mouse model

**Fig 1:**
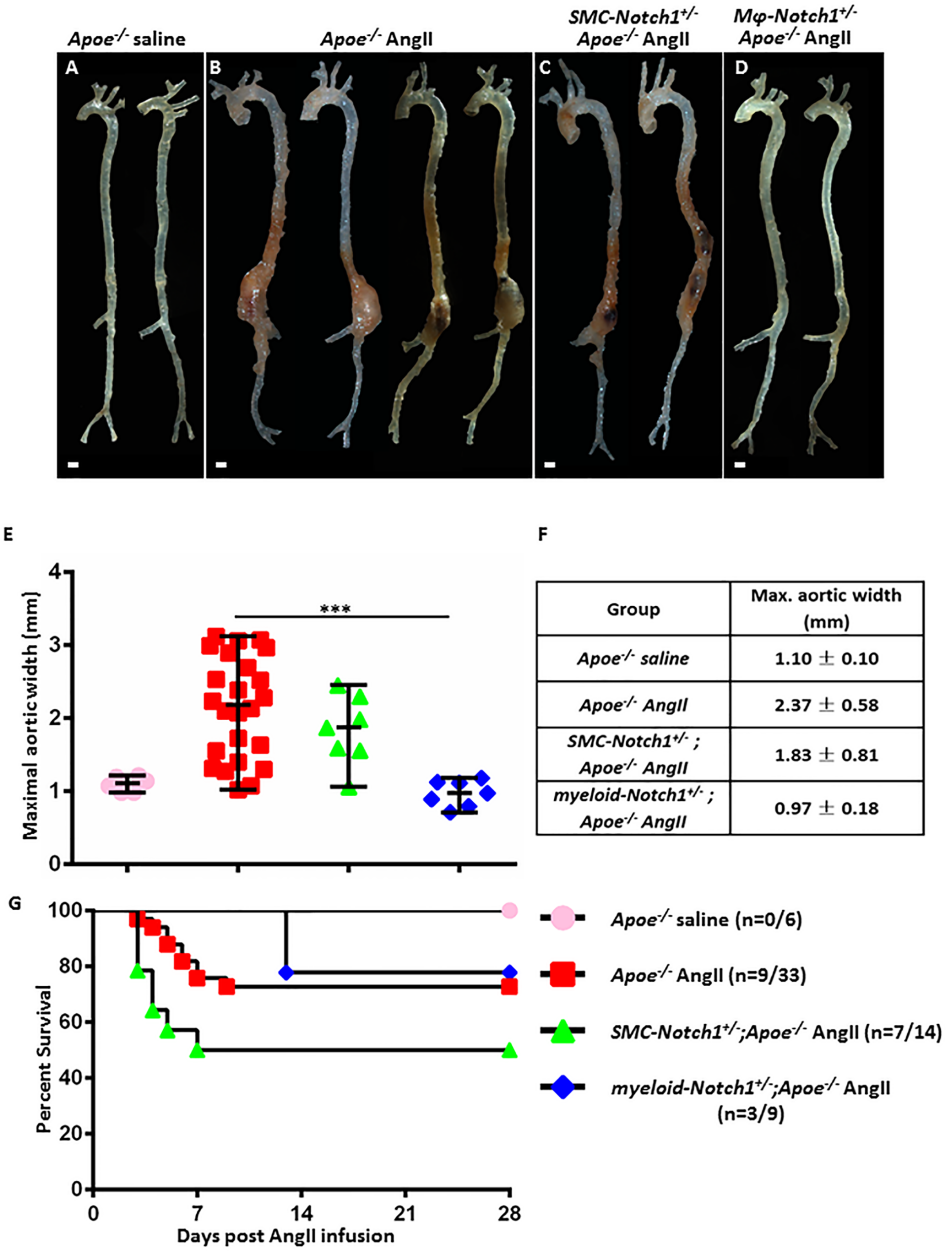
Cell-specific *Notch1* haploinsufficiency differentially interferes with AAA development in AngII-induced mouse model. Representative aortae isolated from *Apoe*^*-/-*^ mice treated with saline (**A**), AngII (**B**), *SMC-Notch1*^*+/-*^*;Apoe*^*-/-*^ mice (**C**;) and *myeloid-Notch1*^*+/-*^*;Apoe*^*-/-*^ mice (**D**;) treated with AngII (28 days). Images were taken using Zeiss Stemi 2000-C microscope. **Scale bar, 1 mm**. (**E-F**), Quantitative measurement of maximal aortic width (mm) in different groups. Each symbol represents an individual animal. Mean and SEM are shown. (**G**) Survival graph showing the mortality of individual mice in each group during 28 day AngII infusion. ***P<0.*001* (post-Bonferroni test with Holm correction).

To determine the contributions of SMCs, ECs, and macrophages in AAA development, we used the Cre-Lox recombination approach. The macroscopic quantification of the external diameter of suprarenal aorta demonstrated a significant exacerbation in maximal aortic width of *Apoe*^*-/-*^ mice with AngII treatment to 2.37 ± 0.58 mm (n = 24, *P*<0.001; Fig 1B, 1E and 1F) compared to saline treated *Apoe*^*-/-*^ mice (1.10 ± 0.10 mm; n = 6; Fig 1A). No difference in the littermate controls was observed in the *Apoe*^*-/-*^ mice for each Cre or *Notch1*^*flox/wt*^ with regard to aneurysm, hence all these mice are pooled in one group (n = 33). The maximal aortic width in *myeloid-Notch1*^*+/-*^*;Apoe*^*-/-*^ mice remained significantly lower as compared to AngII-treated *Apoe*^*-/-*^ mice and was comparable to saline-infused *Apoe*^*-/-*^ mice (0.97 ±0.18; n = 7, *P*<0.001, Fig 1D and 1F). Interestingly, in *SMC-Notch1*^*+/-*^*;Apoe*^*-/-*^ mice, dilation of aorta increased moderately and remained in a narrow range. At day 28 the maximal aortic width in *SMC-Notch1*^*+/-*^*;Apoe*^*-/-*^ mice was modestly but insignificantly lower than AngII-treated *Apoe*^*-/-*^ mice (1.83 ± 0.47; n = 7, Fig 1C, 1E and 1F). The maximal aortic width in EC-*Notch1*^*+/-*^*;Apoe*^*-/-*^ mice was comparable to AngII-treated *Apoe*^*-/-*^ mice (S1 Fig; n = 7). Overall, AngII treatment resulted in 27% mortality in *Apoe*^*-/-*^ mice within 10 days of the treatment (Fig 1G). In S*MC-Notch1*^*+/-*^*;Apoe*^*-/-*^ mice, 50% mortality was observed and occurred within 7 days of treatment, but was not statistically significant as compared to *Apoe*^*-/-*^ mice with AngII treatment (Fig 1G), which may be attributed to either initial influx of Notch1 sufficient macrophages at the vascular lesion or some unrelated effects of *Notch1-*haploinsuffiiciency[44]. In our studies, the aortae from dead S*MC-Notch1*^*+/-*^*;Apoe*^*-/-*^ mice had dissection in the thoracic region. Indeed, various studies have shown strong correlation between Notch1 mutation and thoracic aortic aneurysm (TAA) suggesting that TAA and AAA represent a very complex pathology with regarding to Notch1 signaling pathway[45–47]. Interestingly, the dilation of proximal aorta in the ascending aorta and arch was comparable in *Apoe*^*-/-*^ and S*MC-Notch1*^*+/-*^*;Apoe*^*-/-*^ mice at day 28 of AngII treatment and was larger than in *myeloid-Notch1*^*+/-*^*;Apoe*^*-/-*^ mice (S2 Fig). The trend in the mortality in EC-*Notch1*^*+/-*^*;Apoe*^*-/-*^ mice was similar to S*MC-Notch1*^*+/-*^*;Apoe*^*-/-*^ mice (S1 Fig). The mortality in *myeloid-Notch1*^*+/-*^*;Apoe*^*-/-*^ mice was comparable to *Apoe*^*-/-*^ mice in response to AngII (33%), whereas no deaths were observed in the saline-treated mice (Fig 1G). Summarizing these findings, *Notch1* haploinsufficiency in macrophages is sufficient to prevent the formation of AAA in *Apoe*^*-/-*^ mice in response to AngII, whereas *Notch1* haploinsufficiency in SMCs may interefere with the progression of AAA.

### Cell-specific *Notch1* haploinsufficiency prevents elastin degradation and limits matrix metalloproteinase production

**Fig 2:**
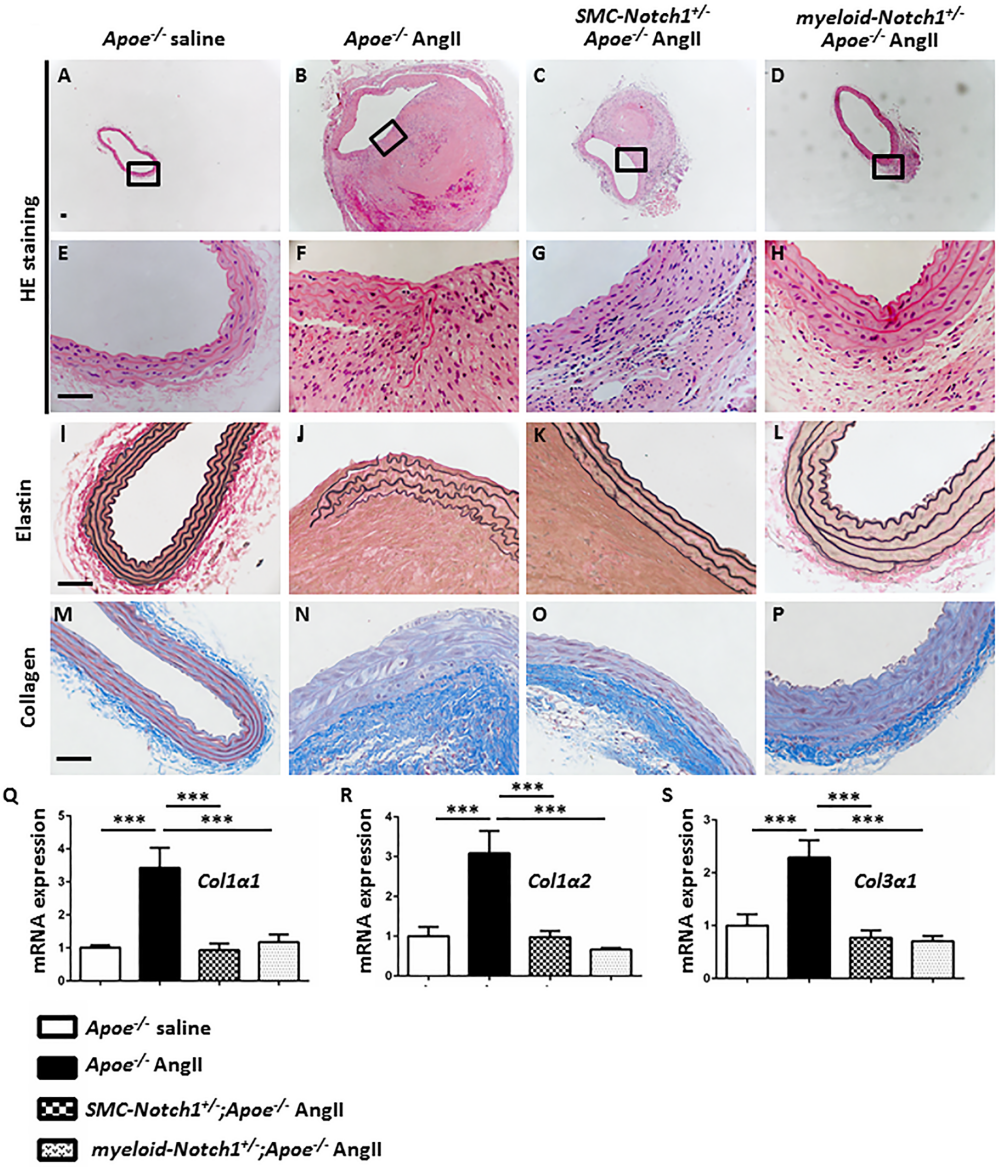
Cell-specific *Notch1* haploinsufficiency in vascular smooth muscle cells and macrophages protects against extensive structural damage and collagen deposition in the abdominal aortic wall in response to AngII (28 days) treatment. **(A-D)** Cross-section of representative aortae stained with hematoxylin and eosin (HE) staining showing lumen and intraluminal thrombus. Panel (**E-H**) Representative photomicrographs showing transmural inflammatory cell infiltration. (**I-L**) Representative images of modified Verhoeff Van Gieson staining demonstrating the extent of elastin fragmentation; (**M-P**) Collagen contents in the abdominal aorta of experimental groups visualized in blue using trichrome staining. **Scale bar = 50** μ**m** in **A-P**. (**Q-S**) Bar graphs represent gene expression of *Col1α1*, *Col1α2 and Col3α1* in the aortae of the *Apoe*^*-/-*^ and cell-specific *Notch1* haploinsufficient *Apoe*^*-/-*^ mice after 28 days AngII treatment. The results were standardized to *Rpl13a* and reported as ratio (mean ± SEM, n = 3 for each group) to saline-treated mice. ***P<0.*001* (ordinary ANOVA followed by a Bonferroni-Holm multiple comparisons test).

The mouse experimental tissues were examined histologically for the characteristic architecture and distinct features of aneurysm. HE staining of the abdominal aorta after 28 days of AngII infusion in *Apoe*^*-/-*^ mice showed dilation of the aortic wall, inflammatory cell infiltration, and disarrangement of ECM with clear signs of elastin fragmentation (Fig 2B, 2F and 2J) as compared to saline-infused *Apoe*^*-/-*^ mice (Fig 2A, 2E and 2I). No such histoarchitectural features of aneurysm were observed in the aortae of myeloid*-Notch1*^*+/-*^*;Apoe*^*-/-*^ mice infused with AngII (Fig 2D, 2H and 2L) which displayed a well-defined lumen, no visible elastin fragmentation and minimal inflammatory cell infiltration. *SMC-Notch1*^*+/-*^*;Apoe*^*-/-*^ mice depicted a distinct morphology of abdominal aorta associated with moderate adventitial thickening, marginal aortic remodeling and no visible signs of elastin fragmentation (Fig 2C, 2G and 2K). Moderate to high collagen content was observed in the adventitial region of abdominal aorta in AngII-treated *Apoe*^*-/-*^ mice (Fig 2N) as compared to *Apoe*^*-/-*^ mice treated with saline (Fig 2M). Collagen content in the adventitial aorta of S*MC-Notch1*^*+/-*^*;Apoe*^*-/-*^ mice were comparable to AngII-treated *Apoe*^*-/-*^ mice (Fig 2O). However, the mRNA expression *Col1α1*, *Col1α2*, and *Col3α1* increased disproportionately by almost 4-fold in response to AngII in *Apoe*^*-/-*^ mice compared with AngII-treated S*MC-Notch1*^*+/-*^*;Apoe*^*-/-*^ mice, suggesting increased collagen degradation and turnover in the former group (Fig 2Q–2S). Collagen content was not considerably altered in the aortae of *myeloid-Notch1*^*+/-*^*;Apoe*^*-/-*^ mice infused with AngII (Fig 2P–2S).

MMPs have been implicated in degrading a wide range of ECM proteins in vascular diseases including AAA. As assessed by the IHC, a high immunoreactivity of Mmp2 and Mmp9 was observed throughout the medial and adventitial layers of abdominal aorta of *Apoe*^*-/-*^ mice with AngII treatment (S3B and S3F Fig) as compared to saline treated *Apoe*^*-/-*^ mice (S3A and S3E Fig). In contrast, minimal Mmp2 and Mmp9 immunoreactivity was present in the abdominal aortae of *SMC-Notch1*^*+/-*^*;Apoe*^*-/-*^ and *myeloid-Notch1*^*+/-*^*;Apoe*^*-/-*^ mice in response to AngII (S3C, S3D, S3G and S3H Fig).

Apoptotic cell death and depletion of medial SMC has been shown to play an important role in human AAA pathological samples and in mouse aneurysmal models[48, 49]. Immunohistochemical analyses showed increased active caspase-3 immunoreactivity in the medial layer of aorta in the *Apoe*^*-/-*^ mice infused with AngII compared with saline treated *Apoe*^*-/-*^ mice (S3I and S3J Fig). Minimal active caspase-3 immunostaining was observed in the abdominal aorta of *SMC-Notch1*^*+/-*^*;Apoe*^*-/-*^ and *myeloid-Notch1*^*+/-*^*;Apoe*^*-/-*^ mice (S3K and S3L Fig). Apoptotic cell death of LPS-stimulated VSMC isolated from the abdominal aorta of *Notch1*^*+/-*^*;Apoe*^*-/-*^ mice was also decreased as compared to *Apoe*^*-/-*^ mice as detected by FACS analysis using annexin V-propidium iodide (PI) staining (S4A–S4C Fig). These data suggest that SMC- and myeloid-specific *Notch1* haploinsufficiency impact the pathological sequelae associated with AAA by different mechanisms. In following experiments, we focused on the mechanism by which SMC-specific *Notch1* haploinsufficiency interferes with the development of AAA.

### Shift in SMC phenotype in SMC-specific *Notch1* haploinsufficient mice is associated with decrease in CTGF expression

Nuclear Notch1 staining representing active NICD was observed in the aortae of *Apoe*^*-/-*^ mice (arrows in S5B Fig). The medial layers of aortae in *SMC-Notch1*^*+/-*^*;Apoe*^*-/-*^ mice were devoid of such nuclear Notch1 staining (arrows in S5C Fig). Surprisingly, no nuclear NICD staining was observed in the medial layer of *myeloid-Notch1*^*+/-*^*;Apoe*^*-/-*^ mice in spite of cytoplasmic presence of NICD (arrows in S5D Fig). The results confirm that depletion of Notch1 signaling reduces the expression of Notch1 activity in respective cell-specific *Notch1* haploinsufficient mice. We previously reported that *Notch1* deficiency protects against AAA development by reducing M1 polarization of Mφ[32, 33]. Increased expression of Irf8 and Mcp1 was observed in the abdominal aortae of *Apoe*^*-/-*^ mice in response to AngII (S5E–S5L Fig). The expression of these cytokines was minimal in the aorta of *myeloid-Notch1*^*+/-*^*;Apoe*^*-/-*^ mice, with concomitant increase in the expression of Cd206. *SMC-Notch1*^*+/-*^*;Apoe*^*-/-*^ mice depicted a distinct phenotype with regard to the expression of macrophage polarization. Whereas, the expression of Irf8 and Mcp1 was comparable to *Apoe*^*-/-*^ mice in response to AngII, the expression of Cd206 was also increased in the adventitia suggesting heterogeneity in the macrophage population (S5G, S5K and S5O Fig). The gene expression of *iNos* (a M1 macrophage marker) was significantly increased in the *Apoe*^*-/-*^ mice in response to AngII compared to other groups, whereas in the *myeloid-Notch1*^*+/-*^*;Apoe*^*-/-*^ mice, expression of *Arg1* (a M2 macrophage marker) was significantly increased compared to the other groups (S5Q and S5R Fig).

**Fig 3:**
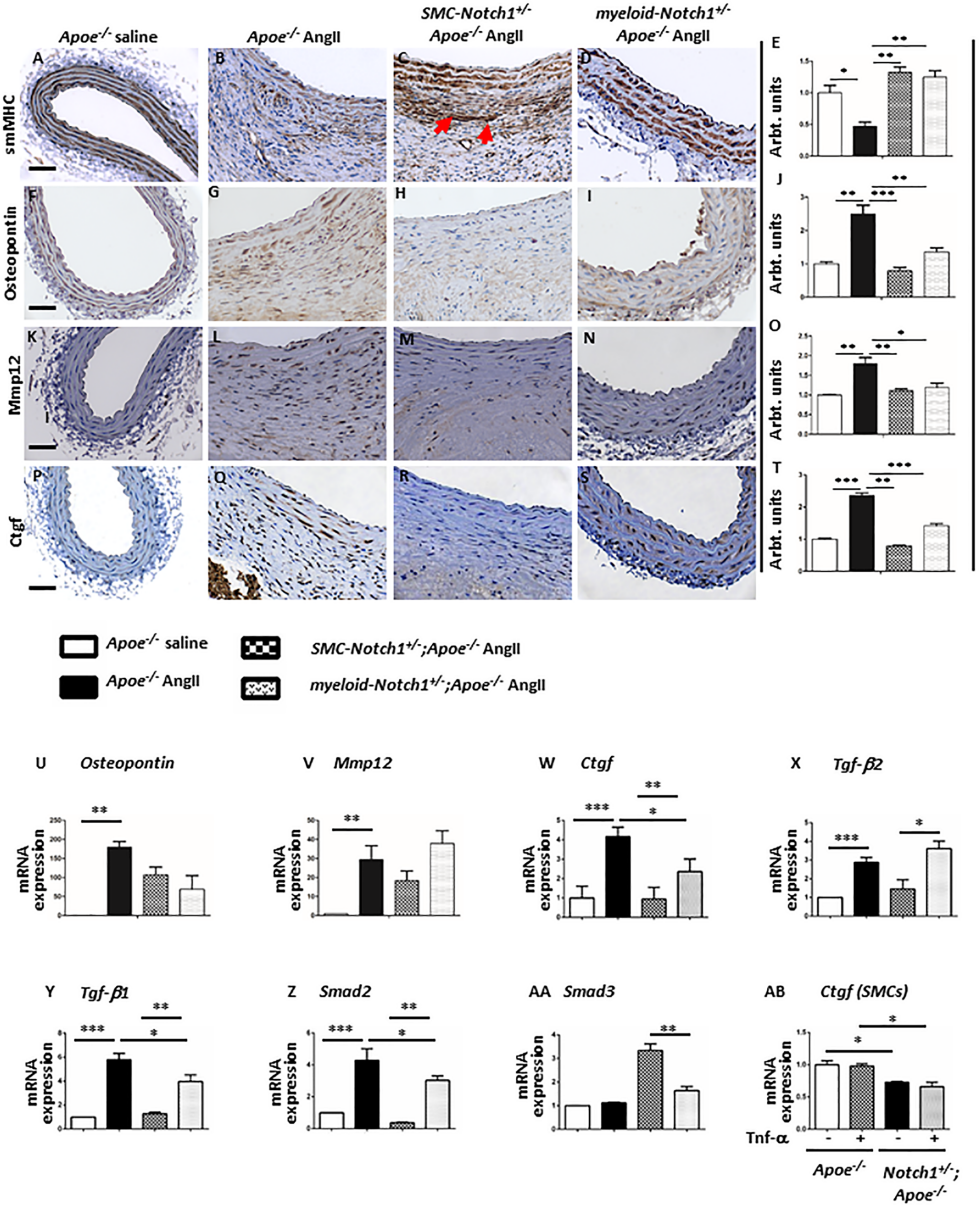
*SMC-specific Notch1* haploinsufficiency dependent regulation of SMC phenotype is associated with changes in CTGF expression. Representative images of IHC staining of abdominal aorta from respective experimental groups with antibodies against smMHC (**A-D**) and osteopontin (**F-I**) as markers of contractile or synthetic-phenotype marker of SMCs, respectively. IHC staining with antibody against Mmp12 (**K-N**), and Ctgf (**P-S**) performed on cross sections of abdominal aortae isolated from *Apoe*^*-/-*^ cell-specific *Notch1* haploinsufficient *Apoe*^*-/-*^ mice after 28 days AngII treatment. **Scale bars = 50** μ**m**. The IHC for respective immunostaining was quantified using ‘Fiji’ version of image J (**E, J, O, T**). Bar graphs represent fold change in gene expression of *Ctgf* (**W**), *Tgf-β1* (**X**), *Tgf-β2* (**Y**), *Smad2* (**Z**) and *Smad3* (**AA**) using mRNA obtained from aortae of *Apoe*^*-/-*^ and cell-specific *Notch1* haploinsufficient mice. The results were standardized to *Rpl13a* and reported as ratio to saline-treated mice (mean ± SEM, n = 3 for each group). (**AB**) Fold change in *Ctgf* expression in SMCs isolated from Apoe^*-/-*^ and *Notch1*^*+/-*^*;Apoe*^*-/-*^ mice at basal levels and in the presence of TNF-α. ***P<0.001; **P<0.01; and *P<0.05 (ordinary ANOVA followed by a Bonferroni-Holm multiple comparisons test).

Next, we examined the factors that define a contractile or synthetic SMC phenotype. Expression of the contractile markers smMHC and SMA-α were highly expressed in the aortae of control *Apoe*^*-/-*^ mice (Fig 3A and S6A Fig). Decreased immunoreactivity for smMHC and SMA-α was observed in the medial layer of abdominal aorta in response to AngII in *Apoe*^*-/-*^ mice (Fig 3B and 3E and S6B Fig). *Notch1* haploinsufficiency in SMCs prevented such loss in the contractile SMC-phenotype markers (Fig 3C, and S6C Fig). Remarkably, the adventitial thickening observed in these groups was also associated with increased expression of smMHC and SMA-α, and was completely absent in the *Apoe*^*-/-*^ mice in response to AngII (red arrows in Fig 3C and 3E and S6C Fig). Expression of contractile markers in the *myeloid-Notch1*^*+/-*^*;Apoe*^*-/-*^ mice was similar to *SMC-Notch1*^*+/-*^*;Apoe*^*-/-*^ mice (Fig 3D and 3E and S6D Fig). However no migration of these contractile SMCs was observed in the adventitia of *myeloid-Notch1*^*+/-*^*;Apoe*^*-/-*^ mice. Conversely, the expression of osteopontin and vimentin, markers of a synthetic-SMC phenotype was increased with AngII infusion primarily in the adventitial region in the aorta of *Apoe*^*-/-*^ mice (Fig 3F and 3J and S6F Fig). The expression of these synthetic-SMC markers was minimal in the abdominal aorta of *SMC-Notch1*^*+/-*^*;Apoe*^*-/-*^ mice. In the *myeloid-Notch1*^*+/-*^*;Apoe*^*-/-*^ mice, the expression of osteopontin appears to be similar to *Apoe*^*-/-*^ mice with AngII infusion. Mmp12 plays an important role in SMC migration and proliferation during vascular remodeling. As shown in Fig 3K–3O, AngII caused induction of Mmp12 expression in both medial and adventitial layers of abdominal aorta in the *Apoe*^*-/-*^ mice compared to control mice. No such increase in Mmp12 expression was observed in the abdominal aortae of *SMC-Notch1*^*+/-*^*;Apoe*^*-/-*^ mice in response to AngII (Fig 3M).

Connective tissue growth factor (CTGF), secreted by synthetic VSMCs and fibroblasts, is a potent ECM–inducing growth factor involved in regulating aneurysm-associated vascular remodeling[50, 51]. We examined if *Notch1* haploinsufficiency in SMCs is associated with alterations in Ctgf expression. Minimal immunostaining of Ctgf was observed in the saline infused *Apoe*^*-/-*^ mice (Fig 3P). In response to AngII, Ctgf immunoreactivity markedly increased in the abdominal aorta of *Apoe*^*-/-*^ mice (Fig 3T) and the distribution was similar to osteopontin and vimentin expression. Moderate expression of Ctgf was also observed in the aorta of *myeloid-Notch1*^*+/-*^*;Apoe*^*-/-*^ mice with AngII treatment (Fig 3Q and 3T). Immunoreactivity of Ctgf was minimal in the aorta of *SMC-Notch1*^*+/-*^*;Apoe*^*-/-*^ mice, suggesting a phenotypic shift with SMC-specific *Notch1* haploinsufficiency (Fig 3R). Next, we examined if Notch1 deficiency causes any alterations in the gene expression of canonical Tgf-β signaling, an upstream regulator of Ctgf pathway. Gene expression of *Ctgf*, *Opn*, *Mmp12*, *Tgf-β1* and *Tgf-β2* was increased in the aorta of *Apoe*^*-/-*^ mice with AngII infusion (Fig 3U–3Y). Myeloid-specific *Notch1* haploinsufficiency did not prevent such an increase in the gene expression of *Ctgf* and *Tgf-β1*. In fact, the expression of *Tgf-β2* was even higher in *myeloid-Notch1*^*+/-*^*;Apoe*^*-/-*^ mice than *Apoe*^*-/-*^ mice with AngII. Conversely, the expression of *Ctgf*, *Tgf-β1* and *Tgf-β2* was significantly decreased in the aortae of *SMC-Notch1*^*+/-*^*;Apoe*^*-/-*^ mice as compared to *Apoe*^*-/-*^ mice (Fig 3W–3Y). Expression of *Smad2*, a downstream target of Tgf-β2 signaling was also increased in *Apoe*^*-/-*^ mice and *myeloid-Notch1*^*+/-*^*;Apoe*^*-/-*^ mice with AngII treatment and was significantly reduced in *SMC-Notch1*^*+/-*^*;Apoe*^*-/-*^ mice (Fig 3Z). Surprisingly, expression of *Smad3* was significantly increased in *SMC-Notch1*^*+/-*^*;Apoe*^*-/-*^ mice (Fig 3AA). The gene expression of Ctgf was also lower in the SMCs isolated from the abdominal aorta of *Notch1*^*+/-*^*;Apoe*^*-/-*^ mice as compared to *Apoe*^*-/-*^ mice at basal conditions or in the presence of TNF-α (10 ng/ml for 24 hours; Fig 3A and 3B).

**Fig 4:**
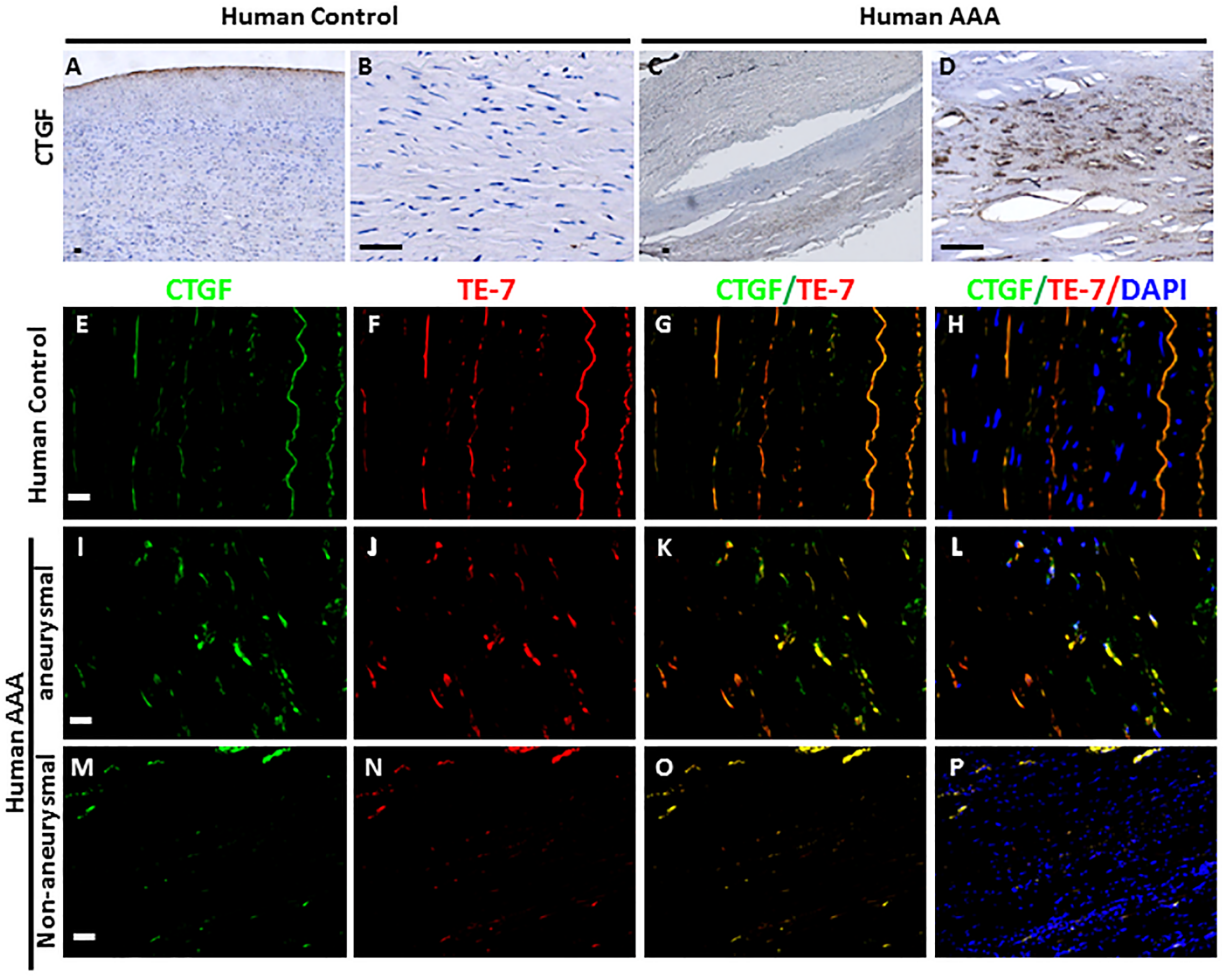
CTGF expression in human AAA correlates strongly with synthetic SMC-phenotype. **(A-D)** Immunohistochemical staining for CTGF on the normal and aneurysmal human abdominal aorta sections. **(E-P)** Micrographs showing immunofluorescence staining for CTGF **(green; E, I, M)** and fibroblast marker TE-7 **(red; E, J, N)** on the tissue sections obtained from medial layer of healthy human controls **(E-H)** or aneurysmal **(I-L)** or non-aneurysmal aorta **(M-P)** from same human AAA. Merged images of CTGF and TE-7 staining from respective tissues **(yellow; G, K, O)** and nuclear staining **(DAPI; H, L, P)** are shown. **Scale bars = 50 μm**.

CTGF has been shown to be a direct modulator of the VSMC phenotype in vascular diseases in human[50]. A significant increase in CTGF-expressing cells was observed in AAA in the inflammatory regions compared with non-inflammatory regions in the same AAA tissue or in the aorta of control human subjects (Fig 4A–4D). The inflammatory and non-inflammatory regions in these tissues were recognized by the presence of macrophages and other inflammatory cells[32]. Next, we examined if increased CTGF expression is associated with synthetic SMC-phenotype in the aorta of human AAA patients. Co-immunostaining with a highly specific TE-7 antibody which identifies fibroblast-like synthetic SMCs[52] suggests that CTGF expression is highly correlated with fibroblast-like synthetic SMC-phenotype (Fig 4E–4P). To further examine if macrophages modulate SMC-phenotype, we performed co-culture assay. The SMCs were obtained from the abdominal aorta of *wild type (WT)* mice and bone marrow derived macrophages (BMDMs) were isolated from either *WT* or *Notch1*^*+/-*^ mice. The BMDMs were pretreated with LPS/IFN-γ to polarize them into pro-inflammatory M1 phenotype as explained [32, 33]. As the data demonstrate, naïve BMDMs from either *WT* or *Notch1*^*+/-*^ mice did not affect the expression of *smMHC*, *Ctgf*, *Mmp12*, or *vimentin* in SMCs. Co-culture with BMDMs, pre-treated with LPS/IFN-γ showed no significant changes in the expression of *Mmp12* or *vimentin*. The expression *Ctgf* and *smMHC*, however was decreased in the *WT* SMCs in response to co-culture with *Notch1*^*+/-*^ BMDM pretreated with LPS/IFN-γ. These data support the hypothesis that Notch1 deficiency may be directly regulating SMC phenotype and that these effects may be mediated independent of its effects on macrophage polarization.

### Gain- and loss-of-function studies suggest direct involvement of Notch1 in regulating CTGF expression

**Fig 5:**
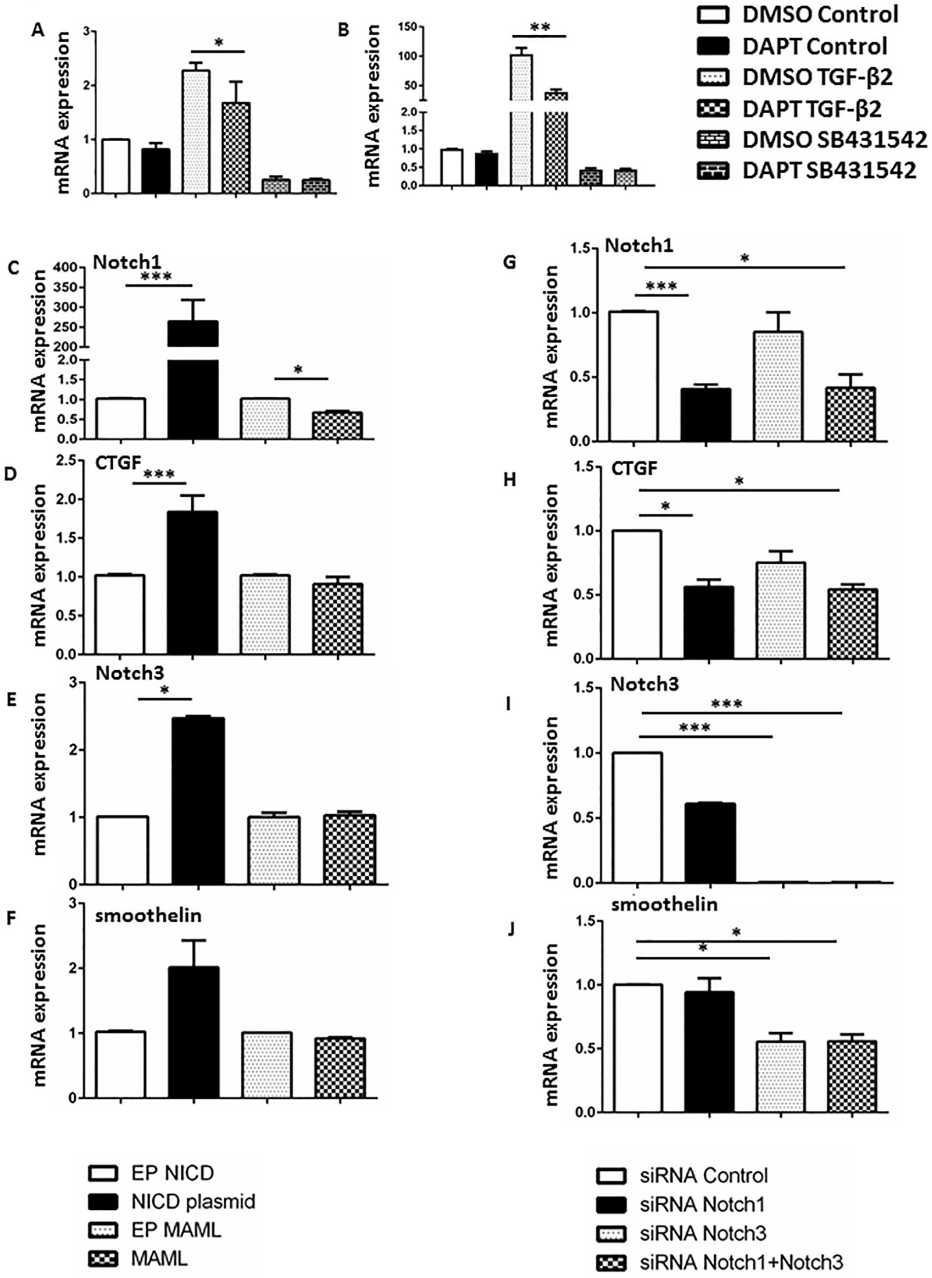
Gain- and loss-of-function studies suggest direct involvement of Notch1 in regulating CTGF expression. **(A-B)** Bar graphs represent fold change in gene expression of CTGF in the human aortic smooth muscle cells **(A)** and mouse embryonic fibroblast **(B)** treated with DAPT for 24 hours followed by TGF-β agonist or inhibitor SB431542 [activin receptor-like kinase (ALK5; the TGF-beta type I receptor inhibitor] treatment for 24 hours. **(C-F)** Graphs represent fold change in the expression of Notch1, CTGF, Notch3 and smoothelin in the human aortic smooth muscle cells 48 hours post transfected with Notch1 *intracellular domain* (NICD) overexpressing plasmid or dominant negative mastermind like proteins (dnMAML). (**G-J)** Graphs represent fold change in the expression of Notch1, CTGF, Notch3 and smoothelin in the human aortic smooth muscle cells 48 hours post transfected siRNAs for Notch1/Notch3.The qPCR data were standardized to *RPL13a* and reported as ratio (mean ± SEM, n = 3 for each group) to empty plasmids for NICD/dnMAML or non-specific siRNA. ***P<0.*001*, **P<0.*01*, *P<0.*05* (ordinary ANOVA followed by a Bonferroni-Holm multiple comparisons test). (EP = empty plasmid).

TGF-β2 is a potent stimulator of SMC phenotypic modulation and is also upstream of CTGF signaling. We determined if Notch1 is upstream regulator in the Tgf-β2 mediated regulation of CTGF. First, we confirmed the expression of CTGF in cultured human aortic SMCs (hASMCs) and mouse primary fibroblasts at basal levels and in response Tgf-β2 agonist or antagonist. At basal conditions, very low expression of CTGF was observed and DAPT (a potent Notch inhibitor) further reduced CTGF expression marginally in these cell types (Fig 5A and 5B). In response to TGF-β2 agonist (human recombinant protein), the CTGF expression increased about 2-fold in hASMCs and 100-fold in mouse fibroblasts. Pre-treatment of hASMCs with DAPT significantly reduced such induction of CTGF expression (Fig 5A and 5B). In contrast, SB431542, an inhibitor of activin receptor-like kinase (ALK5; the TGF- β type I receptor) reduced the expression of CTGF in both cell types ~50% below basal expression. No significant effect of DAPT was observed in response to SB431542 probably because of very low expression of CTGF.

Next, we performed transfection studies on hASMCs using plasmids for NICD or a dominant negative form of the MAML activator (dnMAML) to overexpress or downregulate Notch signaling respectively. NICD-plasmid increased the expression of Notch1 by almost 250-fold and CTGF expression by 2-fold in the hASMCs (Fig 5C and 5D). Conversely, dnMAML-mediated knockdown of Notch signaling did not significantly affect CTGF expression (Fig 5C and 5D). In contrast, siRNA-mediated specific knockdown of Notch1 resulted in a more than 50% reduction in CTGF expression (Fig 5G and 5H). The discrepancy between the dnMAML and siRNA for Notch1 may be attributed to either inhibitory effects of dnMAML on other Notch family members including Notch2 and Notch3, or potential non-canonical activities of Notch1 that are affected by dnMAML. Further, siRNA for Notch3 did not affect the expression of CTGF significantly (Fig 5I). However, decreased expression of smoothelin (a contractile smooth muscle marker) was observed with Notch3 siRNA (Fig 5J). Interestingly, the expression of Notch3 and smoothelin was also increased by almost 2-fold with NICD overexpression (Fig 5E and 5F). No synergistic effects of Notch1 and Notch3 inhibition were observed on the expression of CTGF and smoothelin as determined by the treatment of hASMCs with a combination of siRNA for both Notch1 and Notch3 (Fige 5G–5J). Next, we investigated if the alteration in gene expression of CTGF is translated in the change in protein content. Western Blotting was performed to observe this change. Reduced protein contents of CTGF were observed in *Notch1*^*+/-*^ SMCs and hASMCs treated with DAPT (S8 Fig). In accordance with the gene expression data, Tgf-β2 increased the CTGF protein contents, whereas reduced CTGF contents were observed in hASMCs with SB-431542, an inhibitor of Tgf- β signaling (S8A and S8B Fig). Reduced CTGF content was observed in SMCs with Notch1 deficiency under all these conditions. Overexpression of NICD reversed the protein contents of CTGF in hASMCs (S8C Fig). Collectively, our data suggest that Notch1 may be directly involved in the CTGF-associated hASMC phenotype modulation.

### *Notch1* deficiency reduces the CTGF-induced collagen gel stiffness in VSMCs

**Fig 6:**
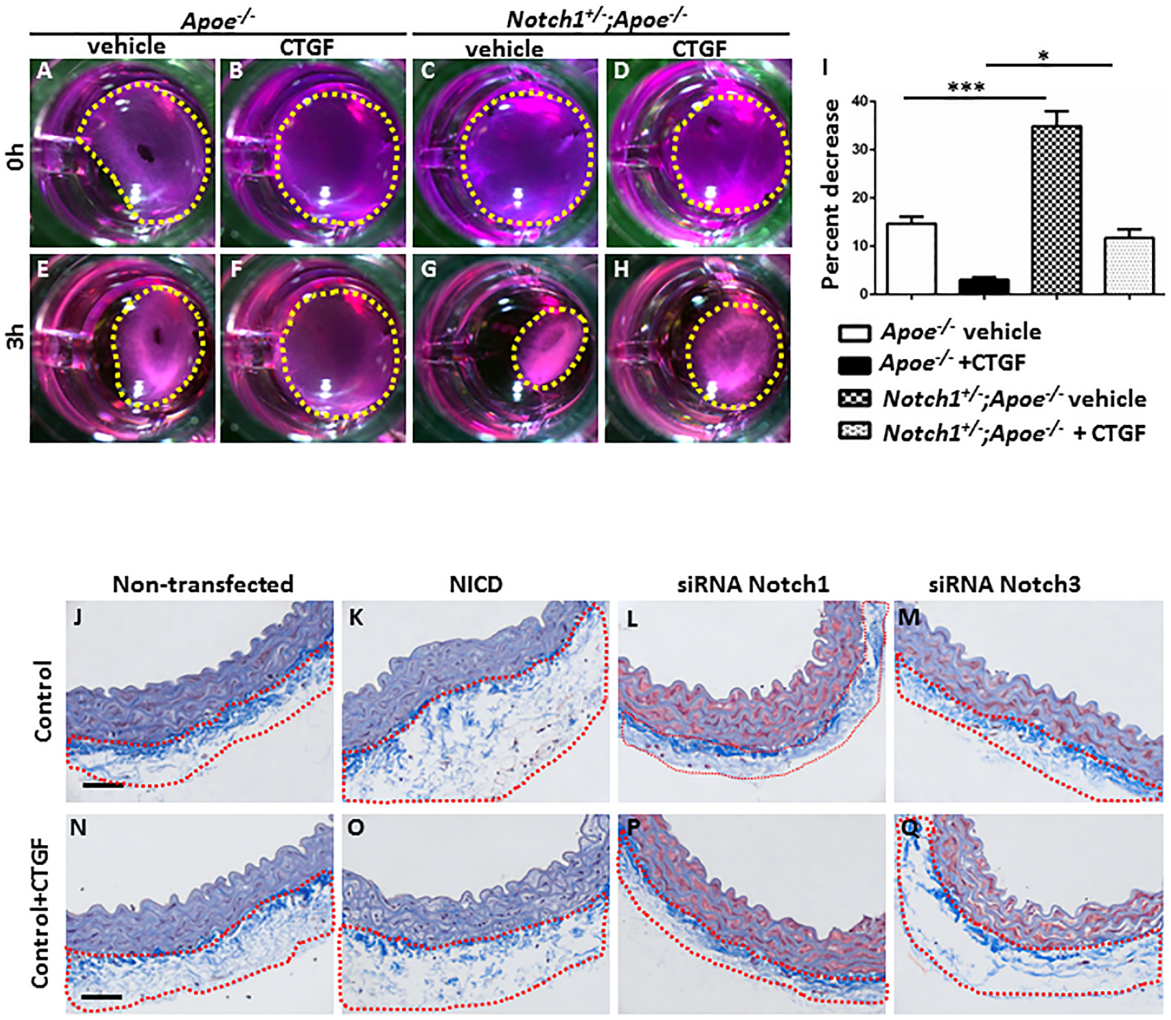
Notch1 deficiency reduces the CTGF-induced collagen gel stiffness in SMCs. **(A-H)** Photographs depict contractility of collagen gel embedded with VSMCs isolated from *Apoe*^*-/-*^ and *Notch1*^*+/-*^*;Apoe*^*-/-*^ mice. VSMCs cultured in a 6-well plate and treated with or without CTGF (10 ng/ml) for 72 hours and resuspended in a collagen solution at a concentration of 6 x 10^5^ cells/ml for 48 hours. After imaging, the gels were gently released from attachment and the media was replaced with 10% FBS DMEM to induce contraction and incubated for 2 hours. The gel was imaged to determine the amount of gel contraction. Each group is represented by 6 replicates in each experiment, and repeated in 3 independent experiments. The images were analyzed using ImageJ software to measure the gel area at each time-point, from which the percentage decrease of gel area was determined. The yellow dotted lines represent the area of the collagen gel. Bar graphs represent percent decrease of gel area in different treatment groups. **(J-Q)** Trichrome staining of the aortic rings from *Apoe*^*-/-*^ mice transfected with NICD, siRNA for Notch1 or Notch3 in the absence **(J-M)** or presence of CTGF **(N-Q)**. The aortic rings were transfected with NICD, non-specific siRNA (4390846, Invitrogen), siRNA Notch1 or siRNA Notch3 for 24 hours. The aortic rings were then treated using recombinant human CTGF protein for additional 48 hours. The rings were then collected and processed for histology. The red dotted lines represent the adventitial layer enriched with collagen contents. **Scale bars = 50 μm**. ***P<0.*001*, *P<0.*05* (ordinary ANOVA followed by a Bonferroni-Holm multiple comparisons test).

Previous studies have demonstrated that Notch1 signaling directly regulates CTGF in osteoclasts which increases fibrotic response[53]. To determine whether CTGF functionally modulates a shift towards a synthetic phenotype, we performed collagen gel contraction assay using VSMCs from the abdominal aortae of *Apoe*^*-/-*^ and *Notch1*^*+/-*^*;Apoe*^*-/-*^ mice. At basal condition, the contraction in the collagen of the VSMCs from *Apoe*^*-/-*^ mice was ~13% in response to 10% FBS (Fig 6A and 6B) whereas the contraction in the collagen of the VSMCs from *Notch1*^*+/-*^*;Apoe*^*-/-*^ mice was significantly higher at ~35% (Fig 6E and 6F; *P*<0.001). The contraction of collagen gel was reduced to just ~5% in the presence of CTGF in *Apoe*^*-/-*^ VSMCs (Fig 6C and 6D), whereas in the VSMCs from *Notch1*^*+/-*^*;Apoe*^*-/-*^ mice, this contraction was significantly higher at ~10% (Fig 6G and 6H; *P*<0.05). We further characterized the inhibitory effect of Notch1 on CTGF-induced changes in VSMC-phenotype using *ex vivo* aortic ring culture from *Apoe*^*-/-*^ mice and NICD plasmid or siRNA constructs[43]. Overexpression of Notch1 increased the collagen content in the adventitial area of aortic rings as compared to the non-NICD transfected aortic rings (marked with red boundary) at basal conditions (Fig 6K) and in the presence of human recombinant CTGF (Fig 6O). Transfection with siRNA Notch1 abrogated such increase in the collagen content at basal levels (Fig 6L) and in response to recombinant CTGF (Fig 6P) suggesting inhibitory effects of Notch1 deficiency on collagen content. Modest increase in the adventitial collagen content was observed in the aortic rings treated with siRNA for Notch3 and CTGF (Fig 6Q) suggesting differential effects of Notch isoforms. No difference was observed in the collagen content in the aortic rings treated with empty plasmid for NICD or non-specific siRNA controls (data not shown).

These findings, coupled with our observations that Notch1 overexpression is correlated with CTGF upregulation, led us to propose that Notch1 signaling is a direct modulator of VSMC-phenotype. These data demonstrate the ability of CTGF to inhibit the contractility behavior of VSMCs and that inhibition of Notch1 signaling prevents such effect in a TGF-β signaling-dependent manner, which may play a role in maintaining VSMCs in contractile phenotype.

## Discussion

**Fig 7:**
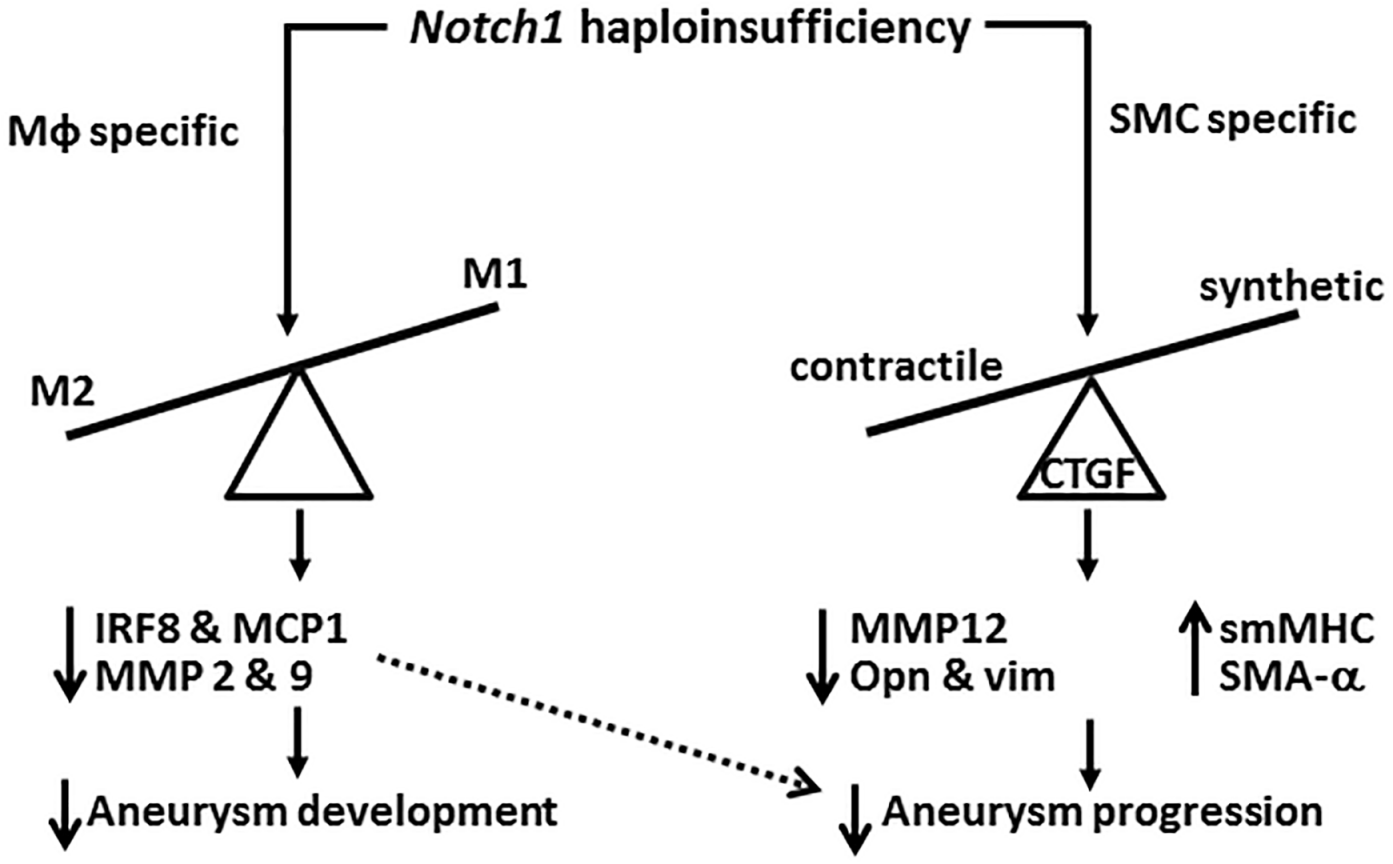
Schematic diagram of the study. The dashed arrows depict the mechanistic pathways to be tested in the study. We propose that myeloid specific *Notch1* haploinsufficiency prevents aneurysm development by modulating macrophage polarization. SMC-specific *Notch1* haploinsufficiency interferes with the progression of aneurysm by modulating CTGF-dependent SMC-phenotype. (Mφ: macrophages, SMC: smooth muscle cells; CTGF, connective tissue growth factor, opn: osteopontin,vim: vimentin).

In the current study, we demonstrated that *Notch1* haploinsufficiency in myeloid cells (LysM-Cre) prevented the formation of AAA and recapitulated histological traits of global Notch1 deficiency including increased M2-differentiation of macrophages and decreased inflammation. Haploinsufficiency of *Notch1* in SMCs (smMHC-Cre) did not prevent the AAA formation in these mice. However, typical histological characteristics of AAA including elastin fragmentation and aortic remodeling were minimal in *SMC-Notch1*^*+/-*^*;Apoe*^*-/-*^ mice as compared to *Apoe*^*-/-*^ mice with AngII. Also, *Notch1* haploinsufficiency in SMCs was associated with increased expression of contractile markers of SMC-phenotype and concomitant decrease in the expression of markers of synthetic SMC-phenotype in the aorta. Expression of CTGF, an ECM-associated protein that modulates synthetic VSMC phenotype, was significantly increased in the abdominal aorta of *Apoe*^*-/-*^ mice in response to AngII. SMC-specific *Notch1* haploinsufficiency prevented such increase in CTGF expression. Increased expression of CTGF was also observed in the adventitial region of the abdominal aorta in human AAA and correlated strongly with synthetic SMC-phenotype. *In vitro* studies on hASMCs and fibroblasts suggest that Notch1 modulates the expression of CTGF by a TGF-β dependent mechanism, which seems to be independent of interactions between SMCs and macrophages. Our data suggest that lack of Notch1 in myeloid cells is sufficient to prevent the formation of AAA whereas SMC-specific *Notch1* haploinsufficiency limits dilation of the abdominal aorta by maintaining contractile SMC phenotype and preventing matrix remodeling (Fig 7).

Extensive studies using mouse models of AAA and our published data using bone marrow transplantation approaches have shown widespread inflammatory macrophages as major cellular components at the onset of the disease[10, 32, 33, 54]. Progression of aneurysm is, however, considered to be interplay between the destruction of elastin and collagen in the media and adventitia and apoptotic cell death of medial SMCs along with thinning of the vessel wall[48]. Some studies have reported that anti-inflammatory interventions in AAA may even accelerate disease progression[55]. However, the specific role of various cell populations of vascular origin in aneurysmal disease remains uncertain. We hypothesize that along with transmural infiltration of inflammatory cells at the early stages of the disease, SMCs contribute to the expansion of aneurysmal growth by participating in the degradation of ECM and increasing the aortic stiffness. Notch1 deficiency in SMCs may interfere with the aortic expansion initiated by the influx of inflammatory macrophages. It is not clear however, if these two factors act independently of each other or macrophages influence SMC by the release of chemokines and cytokines. Some studies have demonstrated that macrophage infiltration induces SMC apoptosis and increased MMP activity, thus contributing to aneurysmal expansion[56]. While our data and other studies have shown that SMCs are independent and actively participate in the process of degrading the aortic matrix[48, 49]. Interestingly, in humans, anti-inflammatory treatment has not been successful in preventing AAA progression making VSMCs an appealing target for further detailed study[57, 58]. Whilst synthetic VSMCs secrete ECM proteins including collagens and elastin, they also secrete MMPs that are involved in ECM breakdown activities[48, 59]. The migration of medial SMCs into intima and media has been shown to play a decisive role in the progression of vascular diseases[60]. Obviously, because of the complexity of AAA pathophysiology and numerous pathways involved in the AAA formation and progression, mere decrease in the inflammatory macrophages may not be sufficient to repair pre-existing aortic dilation and elastin degradation.

Studies have shown that matricellular proteins such as CTGF play an important role in vascular remodeling by regulating ECM deposition, cell proliferation, and de-differentiation of contractile SMC to synthetic SMC phenotype[61]. In human ascending aortic aneurysms, increased reactive oxygen species (ROS) accumulation correlates with media layer degeneration and increased CTGF expression, which modulate the synthetic VSMC phenotype[50]. In addition, CTGF is also involved in monocyte migration and adhesion to injured vessel wall[50, 51]. Our findings are consistent with the previous studies and suggest that CTGF protein is overexpressed in the progression of AAA and correlates with synthetic SMCs[50, 62]. We postulate that Notch1 deficiency in the SMCs decreases the CTGF expression thus protecting against the expansion of aneurysm (Fig 7). Although the mechanism by which CTGF expression is induced in this mouse model is still obscure, possible upregulation of CTGF could be achieved by AngII or by oxidative stress in the medial layers of AAA[50]. Various fibrogenic stimuli including TGF-β, thrombin or mechanical stretch have also been reported to induce CTGF expression. Further studies may be required to define the precise mechanism by which CTGF is activated in the setting of vascular diseases including AAA.

The opposite effects of SMC-specific *Notch1* haploinsufficiency on Smad2 and Smad3 indicate that the crosstalk between Notch1 and Tgf-β signaling pathway in aneurysm may have differential effects on the downstream targets. Indeed, studies have shown that loss of *Smad3* in a mouse model drastically increases wall thickening of the abdominal aorta accompanied by vessel wall remodeling with elastin fragmentation[63]. Studies by Canalis et al[53] have shown that Notch activation directly induces *CTGF* expression in osteoblasts, and that these two pluripotent pathways may act in concert to regulate osteogenic homeostasis. It will be interesting to see if these interactions play a causal role in vascular diseases. In summary, our findings support the hypothesis that CTGF may play a role in AAA pathogenesis by promoting SMC differentiation into proliferative and apoptotic phenotype leading to aortic expansion and thus making CTGF a potential target for future therapies targeting SMC-phenotypic modulation.

Our present work demonstrates that Notch1, not Notch3 is involved in the CTGF-mediated synthetic phenotypic modulation of SMCs. Furthermore, the differential roles of Notch1 in various vascular cells observed in our study demonstrate the complexity of Notch signaling and warrant detailed future studies into the individual contributions of Notch receptors in each vascular cell type on AAA development (Fig 7). Taken together, these data suggest that Notch1 signaling has differential effects on SMC phenotype and provide strong evidence that the phenotypic modulation of SMC-phenotype can influence AAA progression. These studies might prove useful in the future for tailored cell-specific therapy and improve therapeutic efficacy to mitigate the deleterious effects of AAA progression.

## Supporting information

S1 Fig Endothelial cell (EC)-specific *Notch1* haploinsufficiency does not affect AAA development in AngII-induced mouse model. **(A)** Representative images of the cross-section of the abdominal aorta of the EC-specific *Notch1* haploinsufficient *Apoe*^*-/-*^ mice 28 days after AngII treatment. H&E staining showing transmural inflammatory cell infiltration; elastin staining showing fragmentation of the elastin fibers; trichrome staining showing presence of collagen fibers (blue) interspersed within the medial and adventitial layer. Survival graphs represent percentage survival in cell-specific *Notch1*^*+/-*^*;Apoe*^*-/-*^ as compared to *Apoe*^*-/-*^ mice during 28 days AngII infusion. **Scale bar, 1 mm** in **(A)**, 50 **μ**m in **B-E.**
(TIF)

S2 Fig SMC-specific *Notch1* haploinsufficiency does not affect dilatation of proximal aorta in AngII-induced mouse model. Representative aortae isolated from *Apoe*^*-/-*^ mice (**A**); *Mφ-Notch1*^*+/-*^*;Apoe*^*-/-*^ mice (**B);** *SMC-Notch1*^*+/-*^*;Apoe*^*-/-*^ mice **(C);** or EC-*Notch1*^*+/-*^*;Apoe*^*-/-*^ mice **(D)** treated with AngII (28 d treatment). Images were taken using Zeiss Stemi 2000-C microscope. Scale bar, 1 mm. Quantitative measurement of maximal aortic width (mm) of different groups at the ascending aorta **(E)**, aortic arch **(F),** and descending aorta **(G)**. Each symbol represents an individual animal. Mean and SEM are shown. Representative images of the cross-section of the abdominal aorta of different groups 28 d after AngII treatment with H&E staining **(H-S)**. **Scale bar, 1 mm (A-D)**, **50 μm in H-S.**
(TIF)

S3 Fig *Cell-specific Notch1* haploinsufficiency reduces matrix metalloproteinase production and prevents apoptotic cell death. Immunohistochemical staining for Mmp2 **(A-D)**, Mmp9 **(E-H)** and active caspase-3 **(I-L)** performed on cross section of abdominal aortae isolated from *Apoe*^*-/-*^ and cell-specific *Notch1* haploinsufficient *Apoe*^*-/-*^ mice after 28 d AngII treatment. **Scale bars = 50 μm**.
(TIF)

S4 Fig *Notch1* haploinsufficiency prevents LPS-induced apoptotic cell death of SMCs. Cells were grown at ~70% confluence, and treated with LPS (100 ng/ml for 24 h). The cells were collected, stained with annexin V-FITC (5 **μ**l/1x10^5^ cells suspended in 100 **μ**l) in binding buffer for 20 min in the dark, washed 3 times with PBS, and incubated in PI solution (5 **μ**l/100 **μ**l) immediately before 10,000 events were acquired in a Becton Dickinson LSRII flow cytometer **(A, B)**. The percentage of annexin V-positive/PI-negative were quantified with Flow Jo software. Bar graphs show data (mean ± SEM) from three independent experiments **(C)**.
(TIF)

S5 Fig *Myeloid-specific Notch1* haploinsufficiency promotes M2-polarozation of macrophages. Immunohistochemical staining for NICD **(A-D)**, M1 macrophage marker, Irf8 **(E-H)** and Mcp1 **(I-L)** and M2 macrophage marker, Cd206 **(M-P)**. Bar graphs represent fold change expression of *iNos* **(Q)** and *Arg1* (**R**) in the aorta of AngII treated *Apoe*^*-/-*^ and cell-specific *Notch1* haploinsufficient *Apoe*^*-/-*^ mice. The qPCR data were standardized to *Rpl13* and reported as ratio (mean ± SEM, n = 3 for each group) to empty plasmids for NICD/dnMAML or non-specific siRNA. ***P<0.*001*, **P<0.*01*, *P<0.*05*. **Scale bars = 50 μm**.
(TIF)

S6 Fig *SMC-*specific *Notch1* haploinsufficiency preserves contractile-phenotype of SMCs. Immunohistochemical staining for contractile smooth muscle cell (SMC) phenotype marker, smooth muscle-alpha-actin (SMA-α; **A-D**) and synthetic SMC marker, vimentin **(E-H)** performed on cross section of abdominal aortae isolated from cell-specific *Notch1* haploinsufficient *Apoe*^*-/-*^ mice after 28 d AngII treatment. **Scale bars = 50 μm**.
(TIF)

S7 Fig *SMC-*specific *Notch1* haploinsufficiency decreases the expression of CTGF in TGFβ-dependent manner. **(A)** Western blot for Ctgf in WT and *Notch1* haploinsufficient SMCs, **(B)** CTGF protein expression in human aortic smooth muscle cells treated with DAPT for 24 hours followed by TGF-β agonist or inhibitor SB431542 [activin receptor-like kinase (ALK5; the TGF-β type I receptor inhibitor] treatment for 24 hours. (C) CTGF protein expression in human aortic smooth muscle cells 48 hours post transfected with Notch1 intracellular domain (NICD) overexpressing plasmid. GAPDH was used as a loading control for protein normalization in Western blotting in A-C.
(TIF)

S8 Fig Macrophages from either *WT* or *Notch1*^*+/-*^ mice did not influence the expression of synthetic-phenotype of SMCs. Bar graphs represent fold change in gene expression of *Ctgf* (A), *smMHC* (B), *Mmp12* (C), *vimentin* (D) using mRNA obtained from *WT* SMCs co-cultured with *WT* or *Notch1*^*+/-*^ bone marrow derived macrophages (BMDMs) in the absence or presence of LPS/IFN-γ.
(TIF)

S1 Table Schematic presentation to generate cell-specific Notch1 haploinsufficient mice on *Apoe*^*-/-*^ background. (TIF)

S2 Table List of primer sequences used in genotyping (as suggested by Jackson lab). (TIF)

S3 Table Genotyping for cell-specific Notch1 haploinsufficient mice. (TIF)

S4 Table List of antibodies used for immunohistochemistry and immunofluorescence. (TIF)

S5 Table List of primer sequences used in quantitative real-time reverse transcriptase PCR studies. (TIF)
