# Supplementary figures and images for: Smooth muscle cell-specific Notch1 haploinsufficiency restricts the progression of abdominal aortic aneurysm by modulating CTGF expression
Source: PLoS One. 2017 May 31;12(5):e0178538. doi: 10.1371/journal.pone.0178538 (PMC5451061; doi:10.1371/journal.pone.0178538)

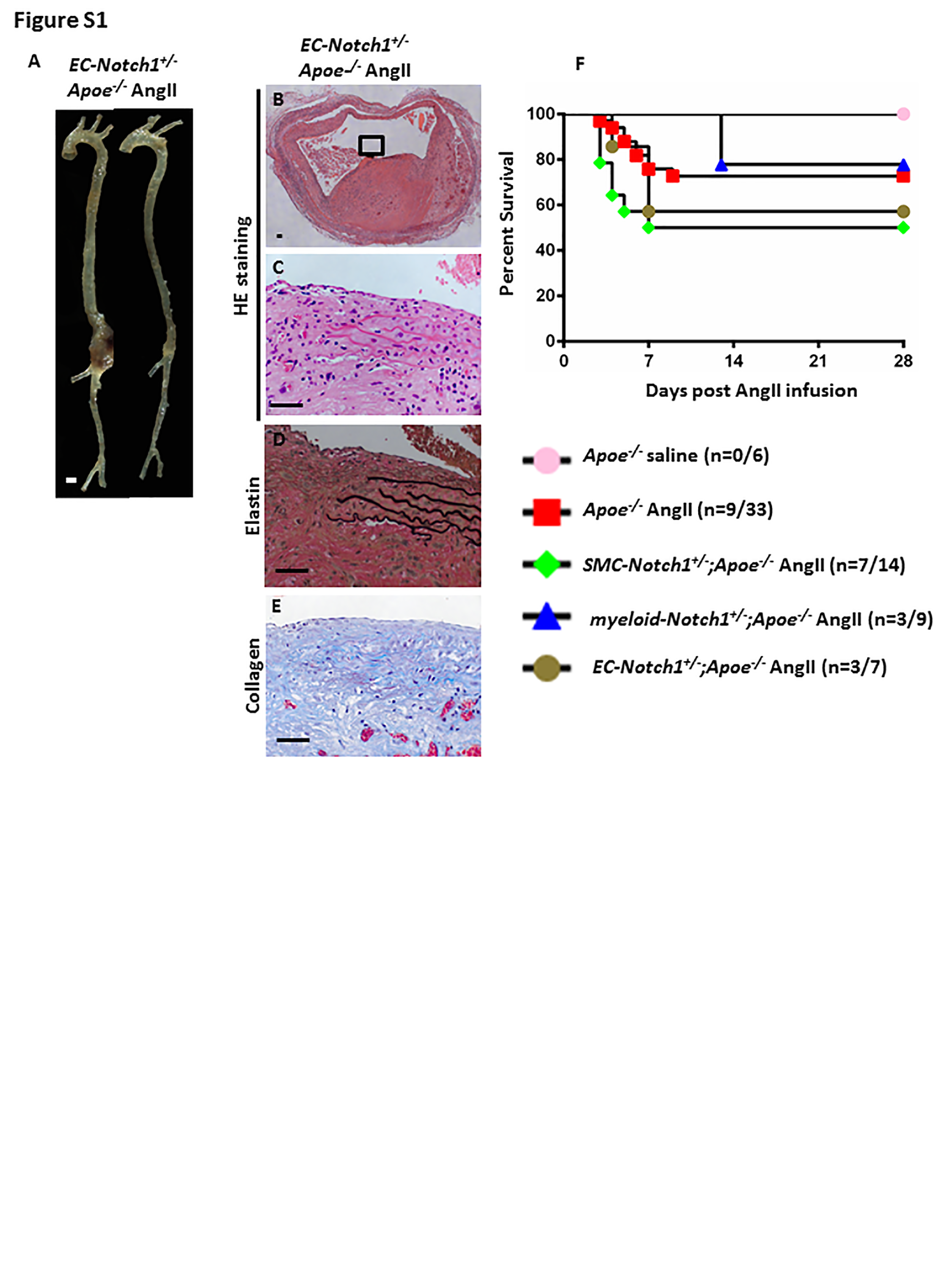

Supplement: S1 Fig — (A) Representative images of the cross-section of the abdominal aorta of the EC-specific Notch1 haploinsufficient Apoe-/- mice 28 days after AngII treatment. H&E staining showing transmural inflammatory cell infiltration; elastin staining showing fragmentation of the elastin fibers; trichrome staining showing presence of collagen fibers (blue) interspersed within the medial and adventitial layer. Survival graphs represent percentage survival in cell-specific Notch1+/-;Apoe-/- as compared to Apoe-/- mice during 28 days AngII infusion. Scale bar, 1 mm in (A), 50 μm in B-E. (TIF) [file pone.0178538.s001.TIF]

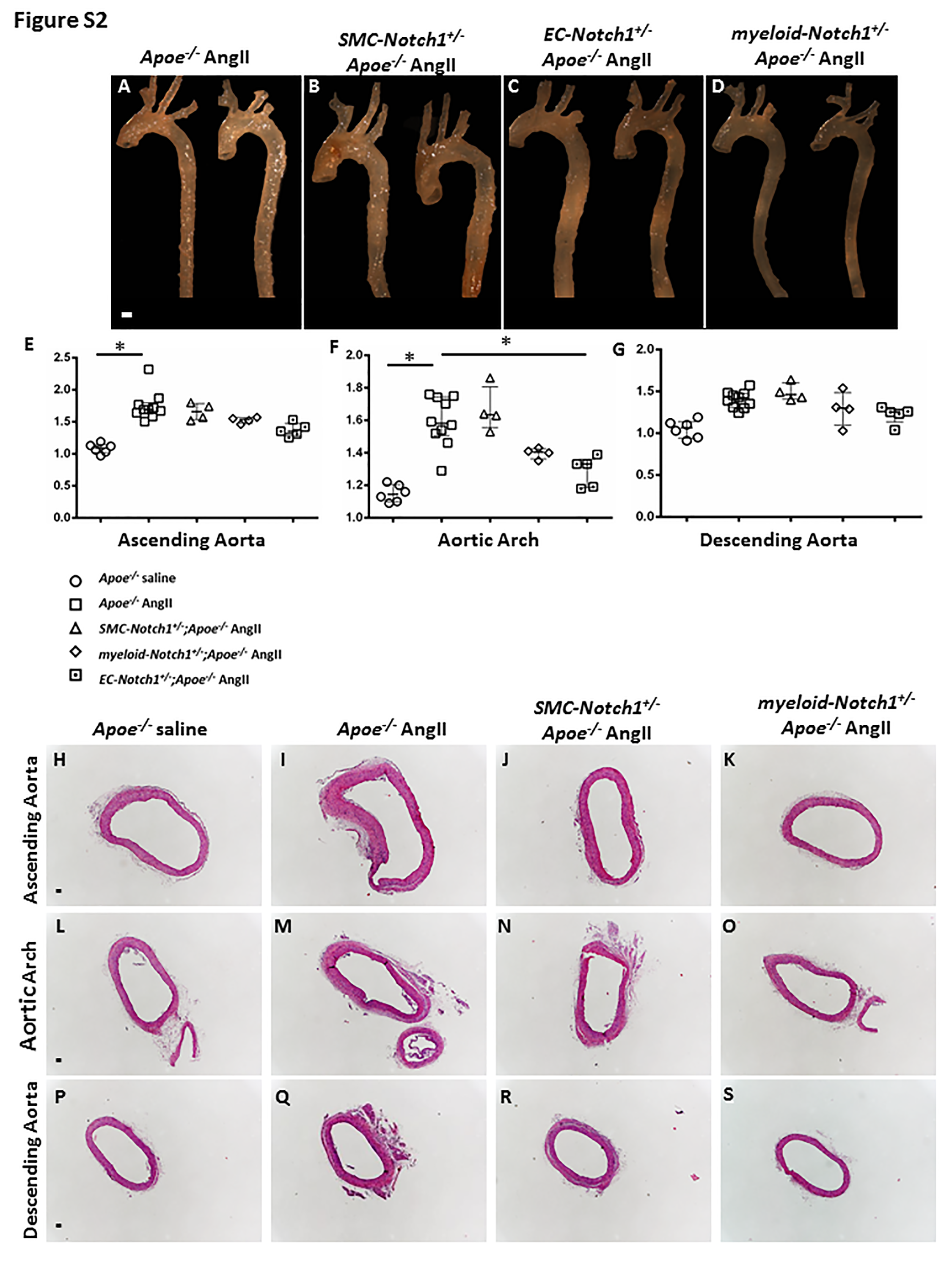

Supplement: S2 Fig — Representative aortae isolated from Apoe-/- mice (A); Mφ-Notch1+/-;Apoe-/- mice (B); SMC-Notch1+/-;Apoe-/- mice (C); or EC-Notch1+/-;Apoe-/- mice (D) treated with AngII (28 d treatment). Images were taken using Zeiss Stemi 2000-C microscope. Scale bar, 1 mm. Quantitative measurement of maximal aortic width (mm) of different groups at the ascending aorta (E), aortic arch (F), and descending aorta (G). Each symbol represents an individual animal. Mean and SEM are shown. Representative images of the cross-section of the abdominal aorta of different groups 28 d after AngII treatment with H&E staining (H-S). Scale bar, 1 mm (A-D), 50 μm in H-S. (TIF) [file pone.0178538.s002.TIF]

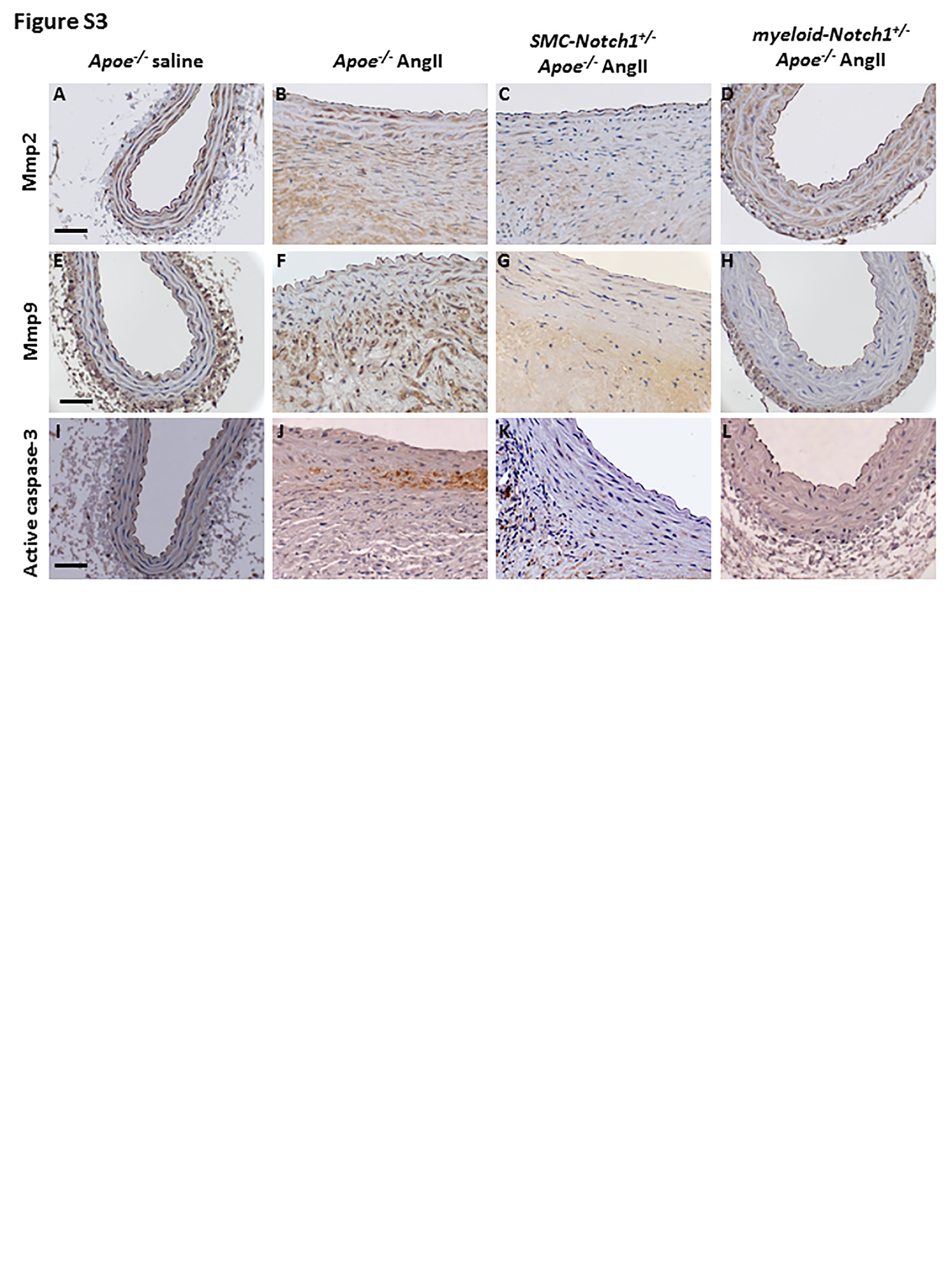

Supplement: S3 Fig — Immunohistochemical staining for Mmp2 (A-D), Mmp9 (E-H) and active caspase-3 (I-L) performed on cross section of abdominal aortae isolated from Apoe-/- and cell-specific Notch1 haploinsufficient Apoe-/- mice after 28 d AngII treatment. Scale bars = 50 μm. (TIF) [file pone.0178538.s003.TIF]

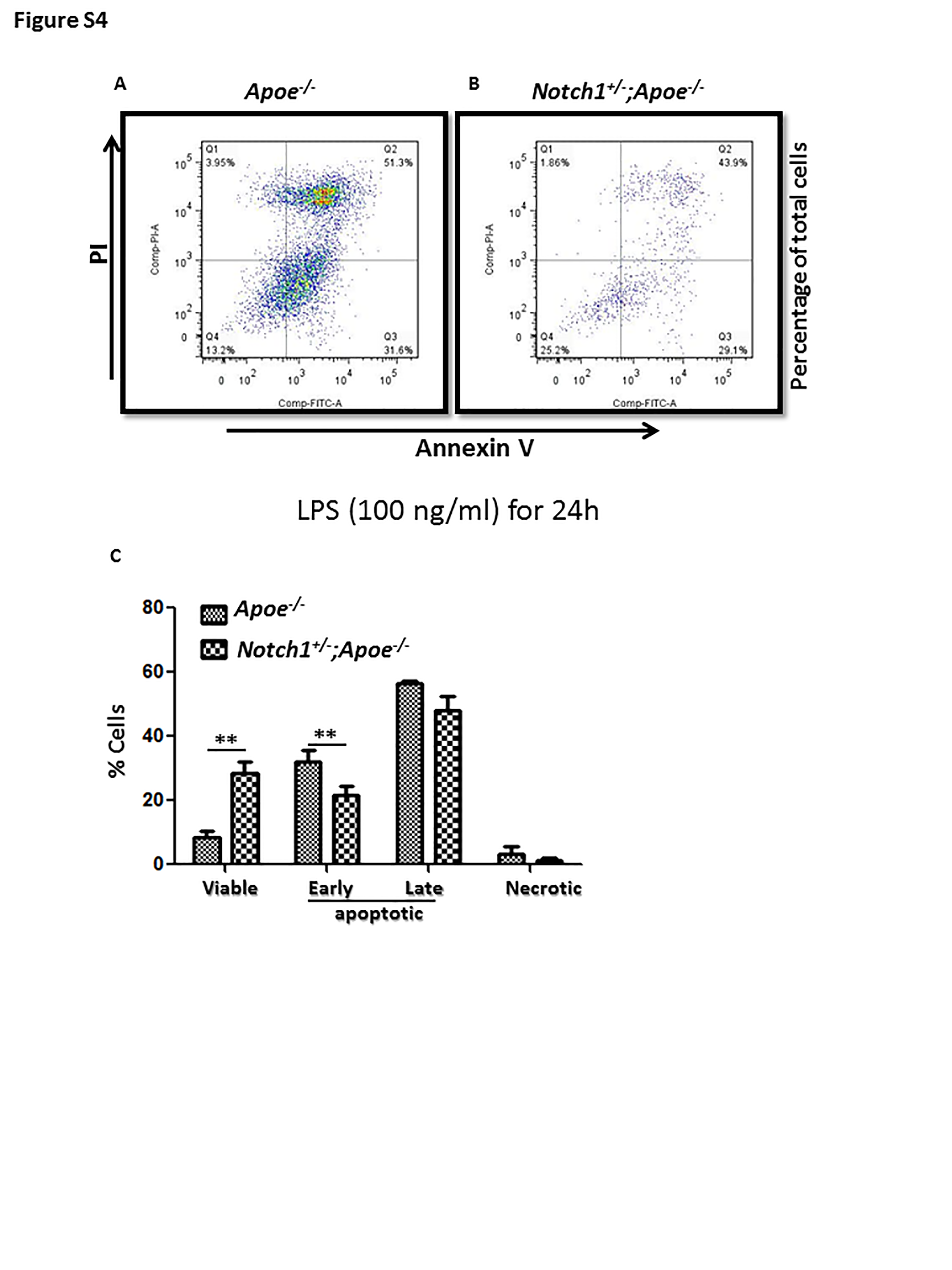

Supplement: S4 Fig — Cells were grown at ~70% confluence, and treated with LPS (100 ng/ml for 24 h). The cells were collected, stained with annexin V-FITC (5 μl/1x105 cells suspended in 100 μl) in binding buffer for 20 min in the dark, washed 3 times with PBS, and incubated in PI solution (5 μl/100 μl) immediately before 10,000 events were acquired in a Becton Dickinson LSRII flow cytometer (A, B). The percentage of annexin V-positive/PI-negative were quantified with Flow Jo software. Bar graphs show data (mean ± SEM) from three independent experiments (C). (TIF) [file pone.0178538.s004.TIF]

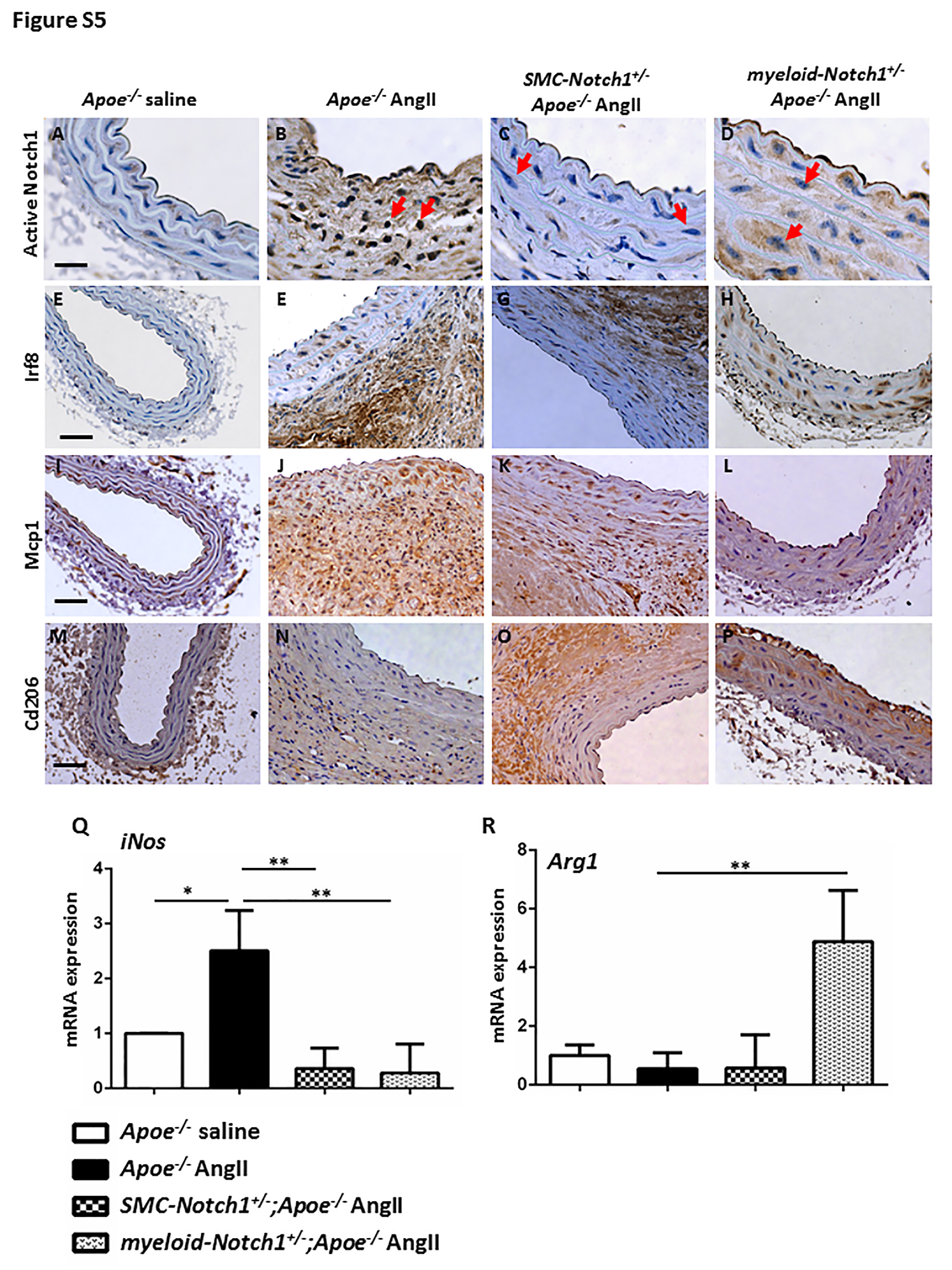

Supplement: S5 Fig — Immunohistochemical staining for NICD (A-D), M1 macrophage marker, Irf8 (E-H) and Mcp1 (I-L) and M2 macrophage marker, Cd206 (M-P). Bar graphs represent fold change expression of iNos (Q) and Arg1 (R) in the aorta of AngII treated Apoe-/- and cell-specific Notch1 haploinsufficient Apoe-/- mice. The qPCR data were standardized to Rpl13 and reported as ratio (mean ± SEM, n = 3 for each group) to empty plasmids for NICD/dnMAML or non-specific siRNA. ***P<0.001, **P<0.01, *P<0.05. Scale bars = 50 μm. (TIF) [file pone.0178538.s005.TIF]

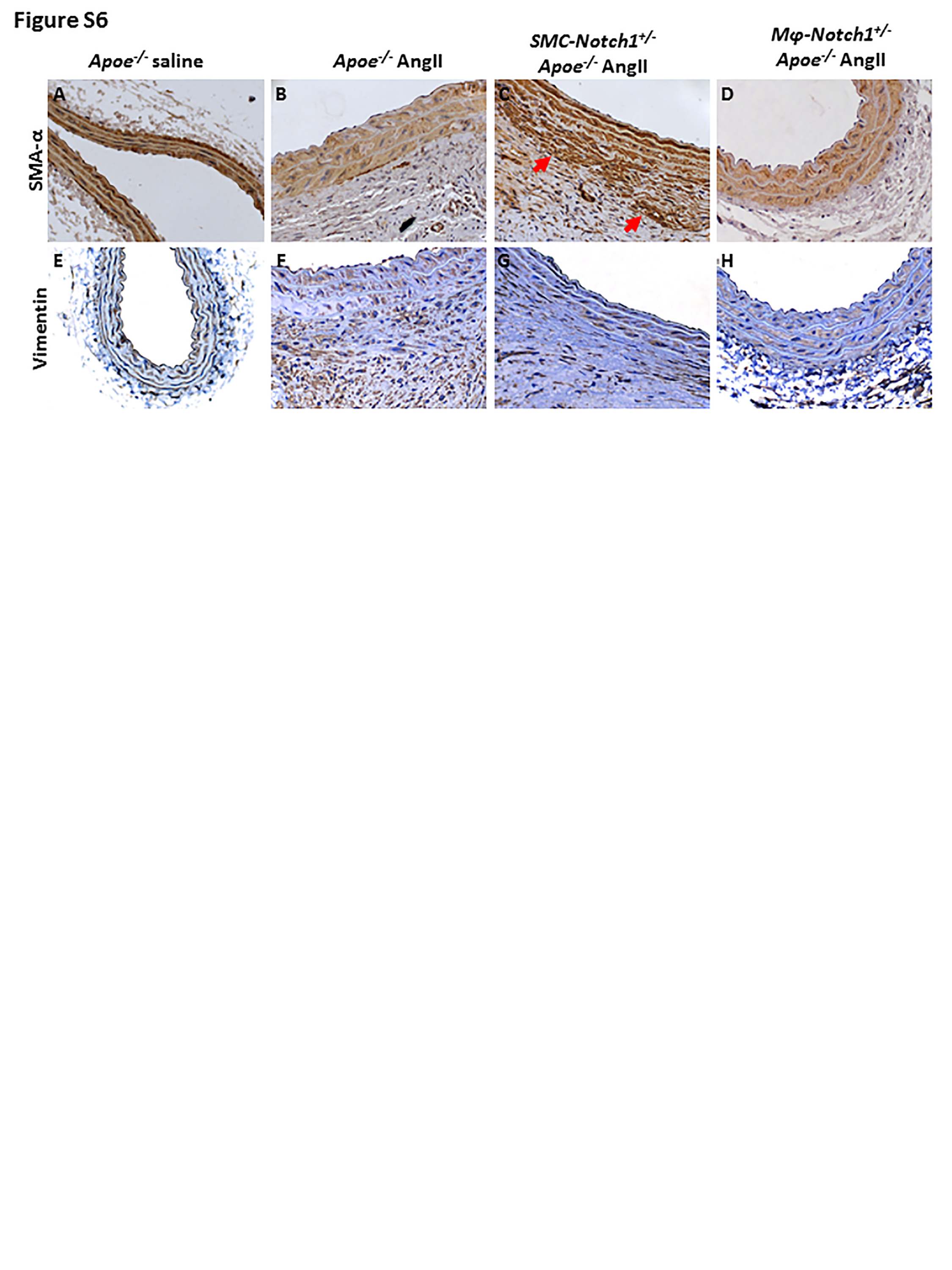

Supplement: S6 Fig — Immunohistochemical staining for contractile smooth muscle cell (SMC) phenotype marker, smooth muscle-alpha-actin (SMA-α; A-D) and synthetic SMC marker, vimentin (E-H) performed on cross section of abdominal aortae isolated from cell-specific Notch1 haploinsufficient Apoe-/- mice after 28 d AngII treatment. Scale bars = 50 μm. (TIF) [file pone.0178538.s006.TIF]

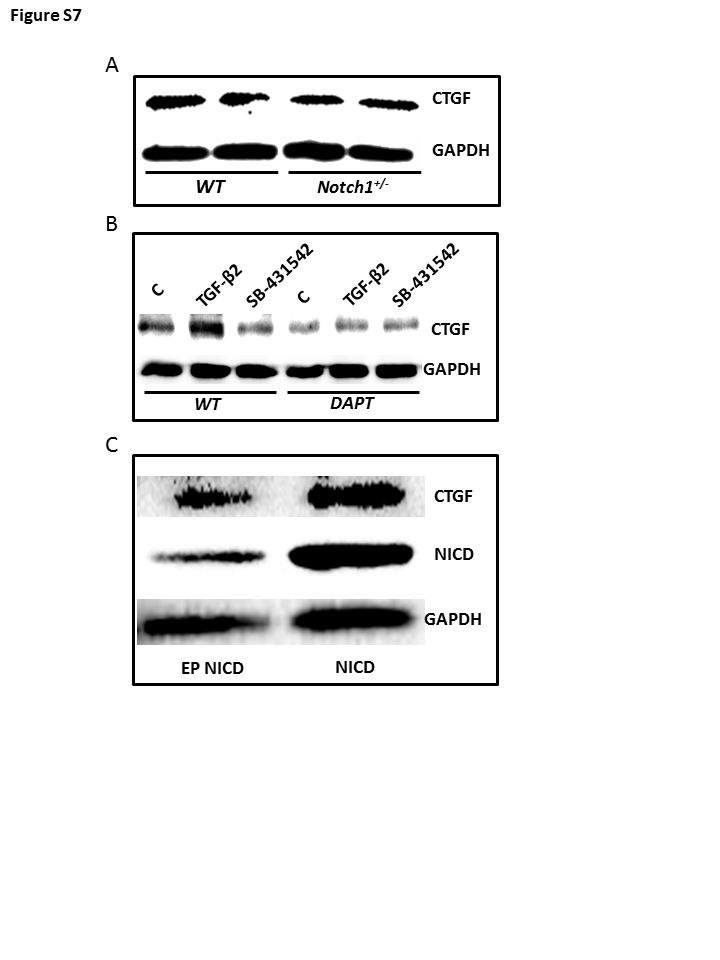

Supplement: S7 Fig — (A) Western blot for Ctgf in WT and Notch1 haploinsufficient SMCs, (B) CTGF protein expression in human aortic smooth muscle cells treated with DAPT for 24 hours followed by TGF-β agonist or inhibitor SB431542 [activin receptor-like kinase (ALK5; the TGF-β type I receptor inhibitor] treatment for 24 hours. (C) CTGF protein expression in human aortic smooth muscle cells 48 hours post transfected with Notch1 intracellular domain (NICD) overexpressing plasmid. GAPDH was used as a loading control for protein normalization in Western blotting in A-C. (TIF) [file pone.0178538.s007.TIF]

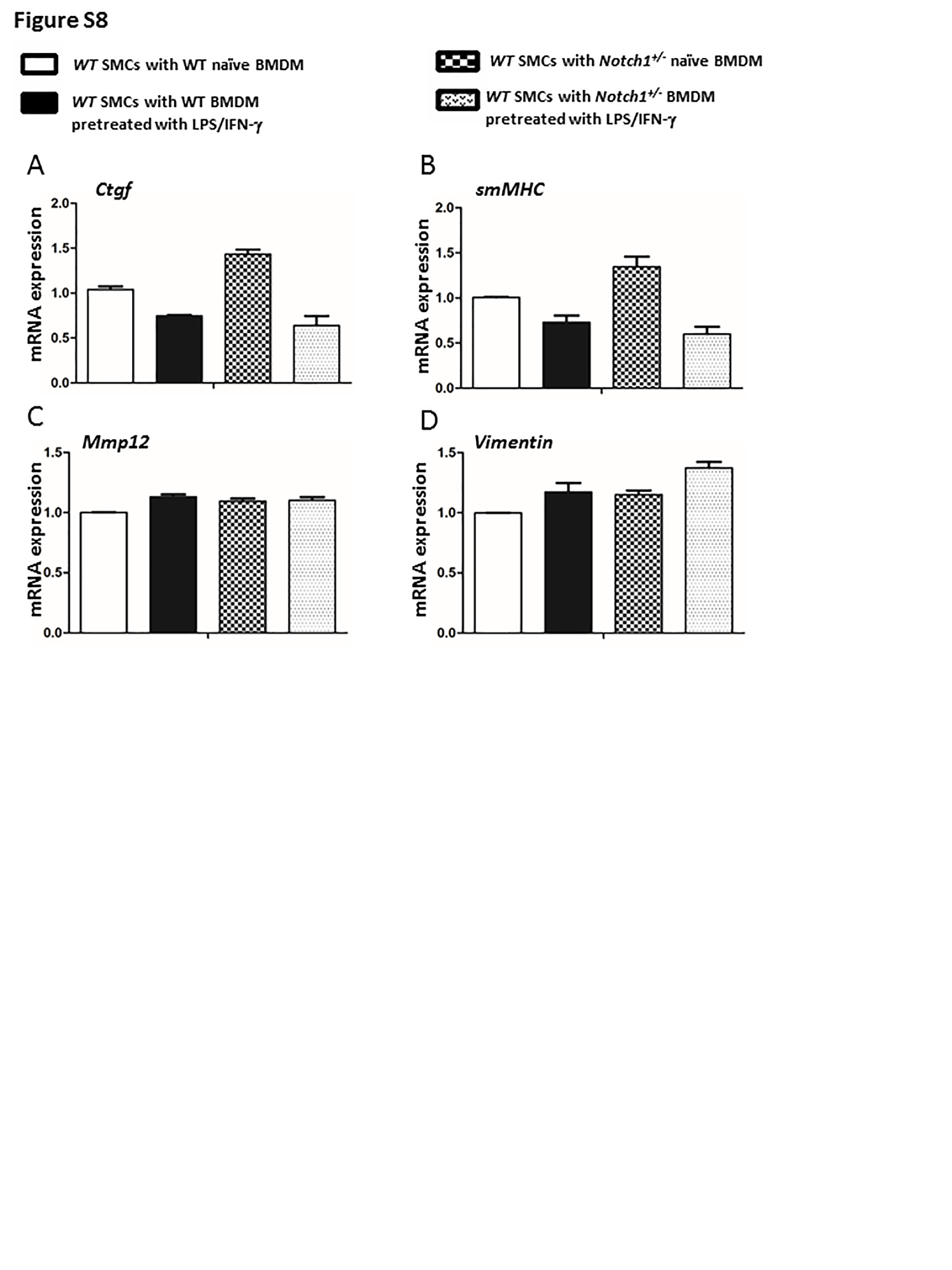

Supplement: S8 Fig — Bar graphs represent fold change in gene expression of Ctgf (A), smMHC (B), Mmp12 (C), vimentin (D) using mRNA obtained from WT SMCs co-cultured with WT or Notch1+/- bone marrow derived macrophages (BMDMs) in the absence or presence of LPS/IFN-γ. (TIF) [file pone.0178538.s008.TIF]

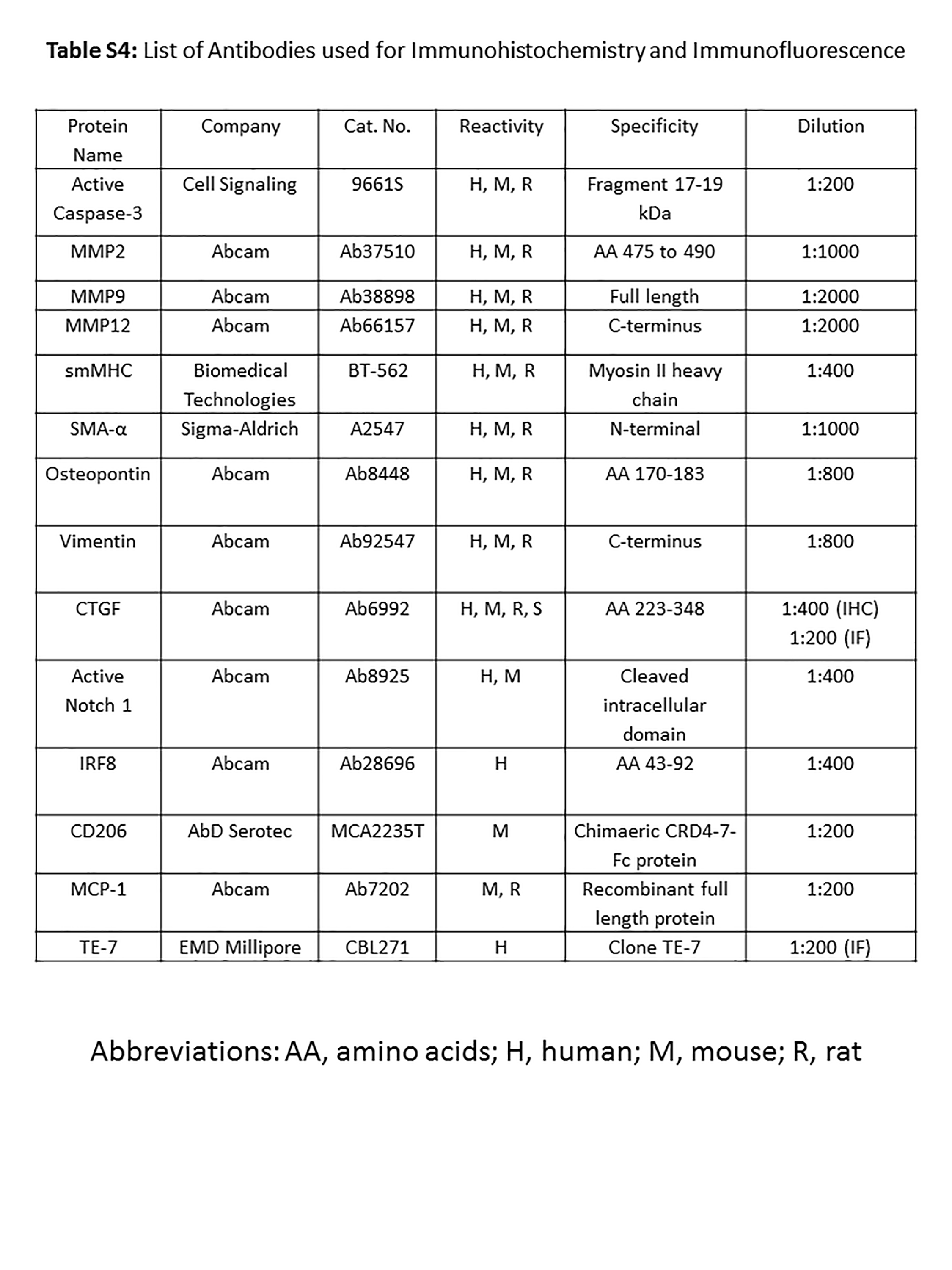

Supplement: S1 Table — (TIF) [file pone.0178538.s009.TIF]

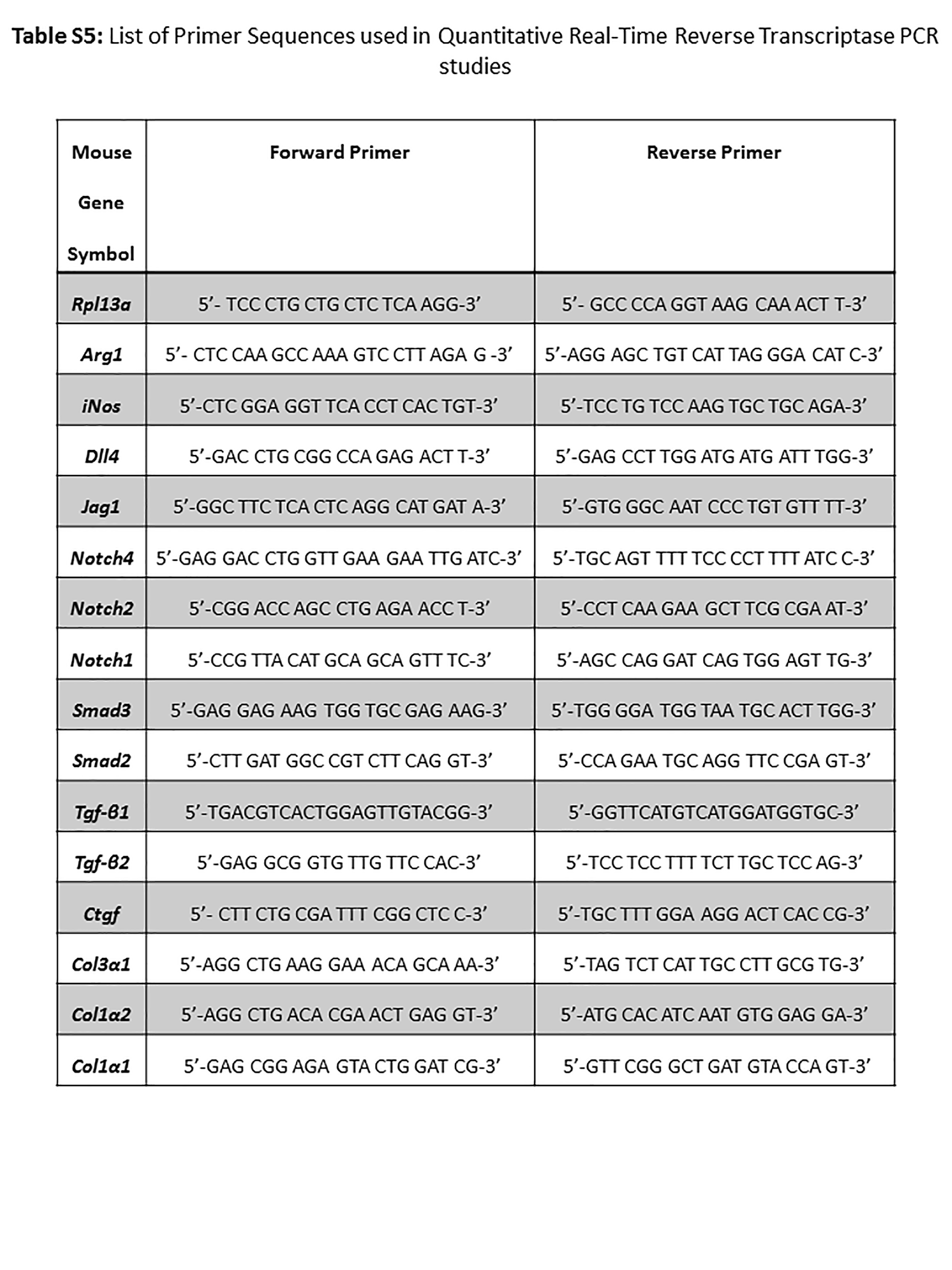

Supplement: S2 Table — (TIF) [file pone.0178538.s010.TIF]

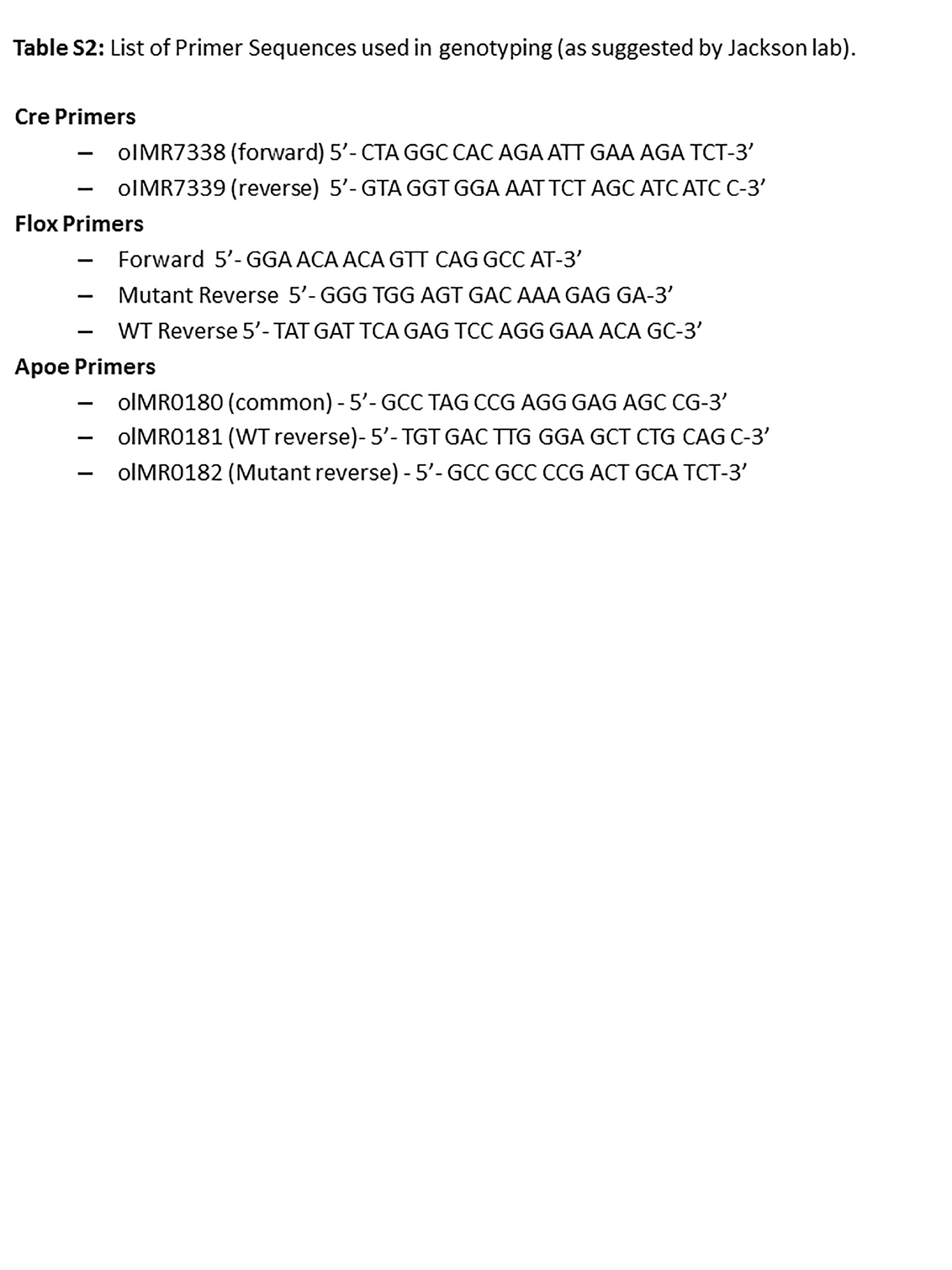

Supplement: S3 Table — (TIF) [file pone.0178538.s011.TIF]

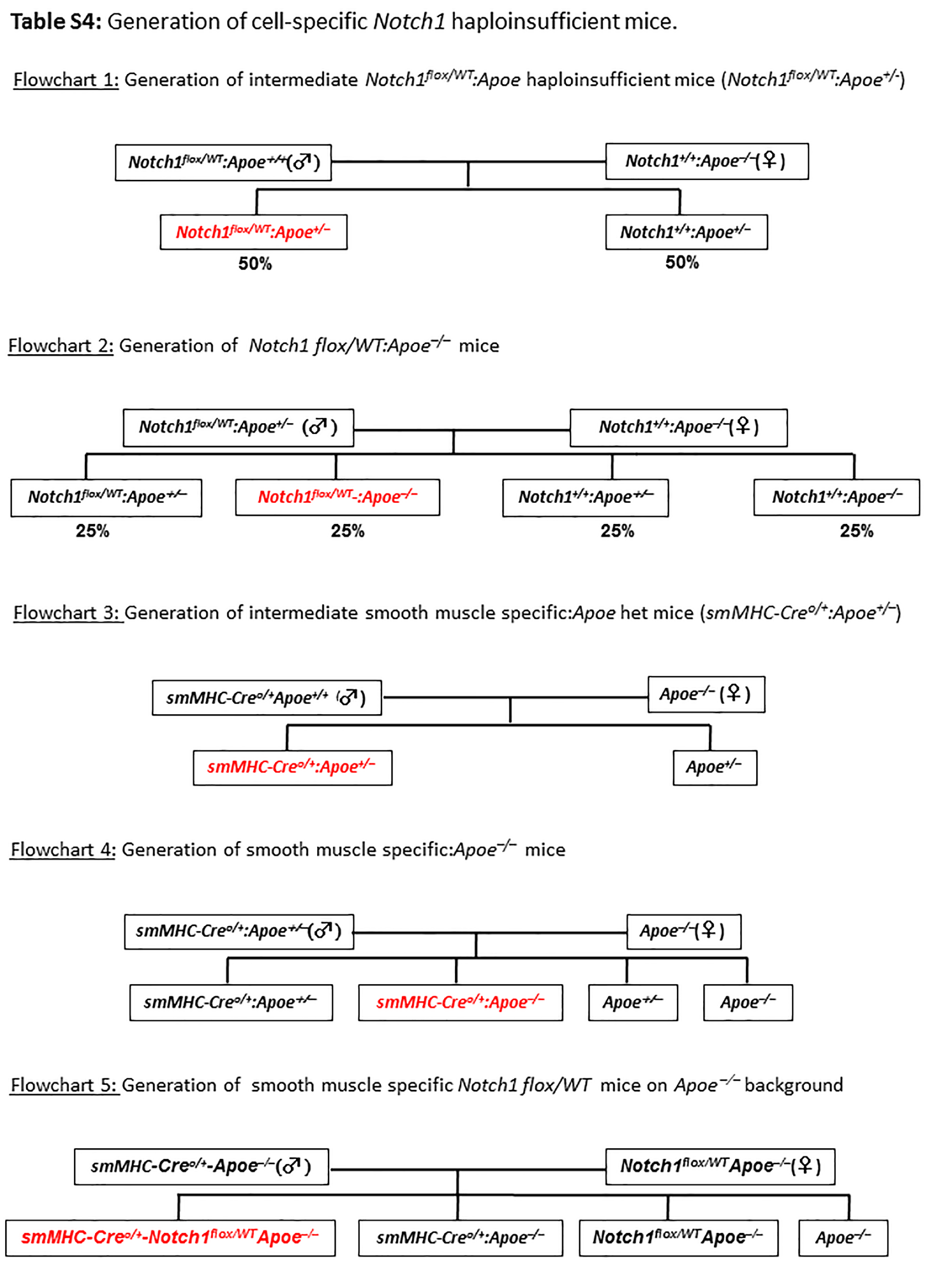

Supplement: S4 Table — (TIF) [file pone.0178538.s012.TIF]

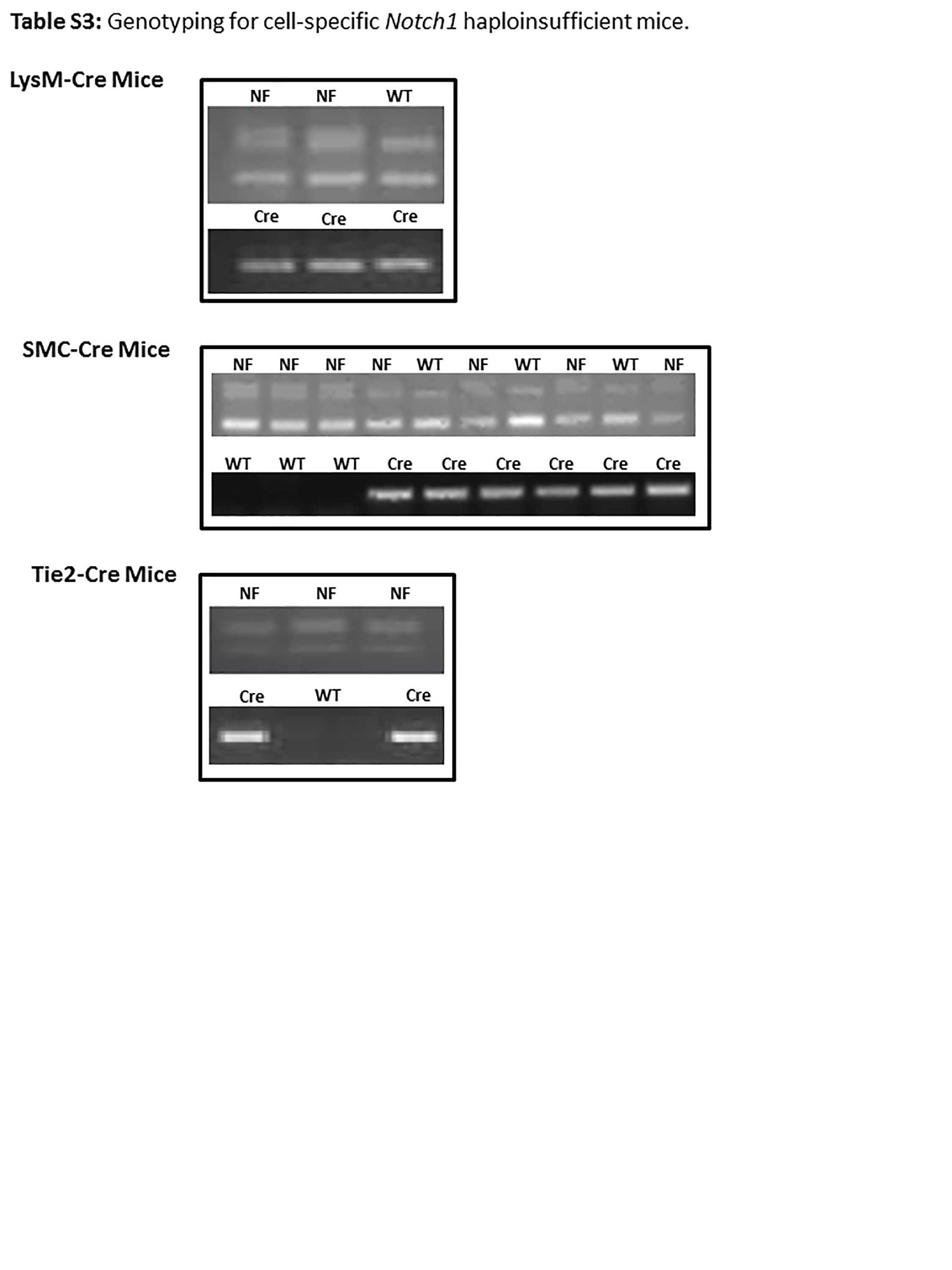

Supplement: S5 Table — (TIF) [file pone.0178538.s013.TIF]
